# Incidence of Adverse Events in Peripheral Intravenous Vasopressor Use: A Systematic Review and Meta-Analysis

**DOI:** 10.1001/jamanetworkopen.2026.0710

**Published:** 2026-03-16

**Authors:** Shang-Jun ZhangJian, Kuang-Yu Niu, Chen-Bin Chen, Chen-June Seak, Chieh-Ching Yen

**Affiliations:** 1 Department of Emergency Medicine, Chang Gung Memorial Hospital, Linkou Branch, Taoyuan, Taiwan; 2 Department of Emergency Medicine, New Taipei Municipal Tucheng Hospital, New Taipei City, Taiwan; 3 College of Medicine, Chang Gung University, Taoyuan, Taiwan

## Abstract

**Importance:**

Peripheral intravenous (PIV) vasopressors are increasingly used but remain associated with adverse events (AEs). Quantifying the incidence of these AEs is essential to guide clinical decision-making regarding PIV vasopressor use.

**Objective:**

To assess the incidence of AEs and avoidance of central venous catheter (CVC) placement after PIV vasopressor administration in adults with hypotension, shock, or critical illness.

**Data Sources:**

PubMed, Embase, and the Cochrane Central Register of Controlled Trials were searched from inception to December 13, 2025, using medical subject headings and keywords related to peripheral vasopressors.

**Study Selection:**

Eligible studies included critically ill adults receiving vasopressors via PIV catheters and reported AEs and/or CVC avoidance.

**Data Extraction and Synthesis:**

Following Preferred Reporting Items for Systematic Reviews and Meta-Analyses guidelines, 2 investigators independently extracted data and assessed risk of bias. A random-effects model was used, and proportions were pooled using a generalized linear mixed model.

**Main Outcomes and Measures:**

The primary end point was the pooled incidence of AEs as the proportion among total catheters, and the secondary end point was the pooled proportion of CVC avoidance.

**Results:**

Forty-nine studies including 33 060 catheters were analyzed. The pooled minor AE incidence was 2.6% (95% CI, 1.4%-4.7%) for norepinephrine, 0.0% (95% CI, 0.0%-24.6%) for epinephrine, 2.9% (95% CI, 1.8%-4.8%) for phenylephrine, 1.4% (95% CI, 0.3%-5.9%) for dopamine, 0.5% (95% CI, 0.1%-1.8%) for vasopressin, and 0.9% (95% CI, 0.1%-11.5%) for metaraminol. When considering all vasopressors collectively, the pooled minor AE incidence was 2.3% (95% CI, 1.5%-3.7%). Regarding major AEs, 30 venous thromboembolism events occurred, all in the 4 studies (8.1%) using midline catheters (1126 total catheters), with a pooled incidence of 1.4% (95% CI, 0.4%-5.4%). In contrast, only 1 major AE (a tissue necrosis event) was reported in 43 studies (87.8%) using short PIV catheters (29 596 total catheters), with a pooled incidence of 0.0% (95% CI, 0.0%-0.0%). CVC avoidance ranged from 0% to 100%, with a pooled proportion of 59.7% (95% CI, 46.4%-71.7%) in 38 studies (77.6%) including 15 371 catheters.

**Conclusions and Relevance:**

In this systematic review and meta-analysis of adult patients, AE incidence was low after short-term vasopressor administration through PIV catheters, particularly short catheters. These findings suggest that PIV administration might reduce the need for CVC placement with appropriate monitoring.

## Key Points

**Question:**

What is the incidence of adverse events (AEs) and avoidance of central venous catheter (CVC) placement after peripheral intravenous (PIV) administration of vasopressors in adults?

**Findings:**

In this systematic review and meta-analysis of 49 studies including 33 060 catheters, pooled incidence of minor AEs for all PIV vasopressors was 2%, while pooled incidence of major AEs was 1% for midline and 0% for short PIV catheters. Pooled incidence of CVC avoidance was 60%.

**Meaning:**

In this study, the incidence of AEs after short-term vasopressor administration through PIV catheters was low, and the findings suggest that with appropriate monitoring, PIV administration might reduce the need for CVC placement.

## Teaser

This systematic review and meta-analysis examines incidence of adverse events and central venous catheter avoidance after peripheral intravenous administration of vasopressors in adults with hypotension, shock, or critical illness.

## Introduction

Shock is defined as inadequate cellular oxygen utilization, resulting in tissue hypoperfusion that, if not promptly identified and treated, can lead to significant morbidity and mortality.^1^ Vasopressors remain a cornerstone in the management of shock. Studies have demonstrated that delays in vasopressor initiation are associated with worse outcomes, including progressive organ failure^2^ and increased 30-day mortality.^3^

Central venous catheters (CVCs) have traditionally been considered the preferred route for vasopressor administration due to concerns about extravasation and the risk of tissue ischemia or necrosis associated with peripheral intravenous (PIV) catheters.^4,5^ However, CVC placement is invasive and carries immediate risks, such as cardiac arrhythmia, vascular injury, and pneumothorax, as well as delayed complications including deep vein thrombosis and bloodstream infections.^6,7,8^ Moreover, inserting a CVC is time consuming and may delay the initiation of vasopressors.^9^

Previous systematic reviews identified a low pooled incidence of predominantly minor adverse events (AEs) from PIV vasopressors^10,11,12^ but lacked agent-specific analyses, data on midline catheters, and estimates of CVC avoidance. To address these gaps and incorporate recent evidence, we conducted an updated systematic review and meta-analysis to characterize AE incidence and assess CVC avoidance in adult patients with hypotension, shock, or critical illness.

## Methods

This systematic review and meta-analysis was conducted in adherence to the Preferred Reporting Items for Systematic Reviews and Meta-Analyses (PRISMA) reporting guideline.^13^ The literature search began in February 2025, whereas data extraction and analysis were conducted after PROSPERO registration in August 2025. Deviations from the registered protocol are described in the eAppendix in Supplement 1. Study selection, data extraction, and quality assessment were independently performed by 2 investigators (S.-J.Z., K.-Y. N.). Discrepancies were resolved by consensus or, when necessary, adjudication by a third investigator (C.-C.Y.).

### Data Sources and Search Strategy

We conducted a comprehensive literature search of 3 electronic databases—PubMed, Embase, and the Cochrane Central Register of Controlled Trials (CENTRAL)—to identify eligible studies from inception to December 13, 2025. The search strategy used both medical subject headings and keywords related to peripheral vasopressors, as detailed in eTable 1 in Supplement 1. No restrictions were placed on language, country of publication, or publication date. In addition, we performed a manual search of the reference lists of all included studies and relevant review articles to identify additional eligible studies.

### Definition and Study Selection

Our primary end point measured the pooled incidence of localized anatomic complications associated with PIV vasopressor administration, stratified by minor AEs and major AEs. We calculated the proportion by dividing the number of local anatomic complications by the total number of catheters that received PIV vasopressor administration. The secondary end point was the pooled proportion of catheters for which CVC placement was avoided through PIV vasopressor administration. A vasopressor was defined as any medication that induces vasoconstriction and causes an elevation in blood pressure. Minor AEs included pain at the injection site, local tissue swelling or edema, infiltration, extravasation, tissue cannulation, cellulitis, and thrombophlebitis, which require only conservative management. Major AEs included skin or tissue necrosis, limb ischemia, venous thromboembolism (VTE), and events requiring surgical intervention such as debridement or amputation, as in previous studies.^10,11,14,15^ A PIV catheter was defined as an intravenous catheter terminating in a peripheral vein, typically in the arm or leg. We also included midline catheters as PIV catheters since they function similarly.^16,17^ A CVC refers to a catheter placed into the central venous system, usually in the internal jugular, subclavian, or femoral veins.

Two investigators (S.-J.Z., K.-Y.N.) independently screened each study for eligibility. Studies were deemed eligible for inclusion if they met the following criteria: (1) adult patients (18 years or older); (2) patients with hypotension, shock, or critical illness or those at risk of shock requiring vasopressors; (3) administration of vasopressors through PIV catheters, including midline catheters; (4) reporting of any AEs and/or the proportion of CVC avoidance associated with PIV vasopressor infusion; and (5) randomized clinical trials (RCTs), observational cohort studies (prospective or retrospective), or case series with at least 10 patients. We excluded duplications, case reports, pediatric studies, conference abstracts, and editorials. Titles and abstracts of all retrieved articles were first screened for potential relevance by the 2 investigators. Full-text screening was subsequently performed for studies meeting the inclusion criteria. Discrepancies were resolved through discussion or, if necessary, by consulting a third investigator (C.-C.Y.). To evaluate the reliability of the eligibility assessments, interobserver agreement between the 2 investigators was quantified using the Cohen κ statistic.

### Data Extraction and Quality Assessment

To ensure consistency, data extraction was conducted independently by 2 investigators (S.-J.Z., K.-Y.N.) using a standardized form in Excel LTSC 2021 (Microsoft Corporation). When study details were incomplete or ambiguous, the corresponding authors were contacted for clarification. The extracted dataset captured key study characteristics, including authorship, year, design, geographic location, eligibility criteria, and clinical setting.

For risk-of-bias assessment, we used 2 validated tools: the Joanna Briggs Institute (JBI) checklist for prevalence studies^18^ and the revised Cochrane risk-of-bias tool (RoB 2)^19^ for RCTs. Since the JBI checklist does not provide operational definitions for categorizing risk-of-bias levels and only yields an overall score, we predefined a classification scheme based on score tertiles: the top tertile was designated as low risk of bias, the bottom tertile as high risk of bias, and the middle tertile as unclear risk of bias.

### Statistical Analysis

We used a random-effects model and pooled proportions using a generalized linear mixed model with Clopper-Pearson intervals.^20^ The random-effects model was predetermined for analyses due to expected heterogeneity in study methods, clinical setting, geographic locations, and participant demographics. Between-study heterogeneity was assessed using the Cochran *Q* test and *I*^2^ statistic, with considerable heterogeneity defined as *I*^2^ ≥ 75%.^21^

To explore the factors that might contribute to the expected variability in the proportion of AE and CVC avoidance across studies, we conducted prespecified subgroup analyses based on the following study characteristics: publishing year (>2021 or ≤2021), study design (prospective study, retrospective study, or RCT), study setting (operating room [OR], intensive care unit [ICU], ward, emergency department [ED], or mixed), duration of infusion (≥24 or <24 hours), PIV gauge (≥22 or <22 G), PIV location (including and above antecubital fossa, below antecubital fossa, or other), protocol use (yes, no), and type of PIV catheter (midline, short). The year 2021 was chosen a priori as a cutoff because in that year, the Surviving Sepsis Campaign recommended initiating vasopressors peripherally in adults with septic shock rather than delaying until central venous access is secured.^22^ We performed 2 sensitivity analyses to recalculate the pooled estimates: first by excluding studies with a high risk of bias and second by using the leave-1-out method to assess the influence of each individual study. Publication bias evaluation used Doi plot visualization and the Luis Furuya-Kanamori (LFK) asymmetry index,^23^ providing quantitative asymmetry assessment with the following interpretation: values of 1 or less represent no asymmetry, values greater than 1 to less than 2 suggest minor asymmetry, and values of 2 or greater indicate major asymmetry. Additionally, we applied Peters and colleagues’^24^ regression method to examine the association between SE and effect magnitude across included studies. Certainty of evidence was not assessed using Grading of Recommendations, Assessment, Development, and Evaluations (GRADE), as its application to systematic reviews of prevalence outcomes remains insufficiently developed and validated.^25^ Statistical analyses and meta-analyses were performed using R, version 4.3.2 (R Project for Statistical Computing), with the metaprop and metasens packages. All statistical tests were 2-sided. The significance level was set at *P* < .05, and 95% CIs were reported.

## Results

### Search Results

**Figure 1.**
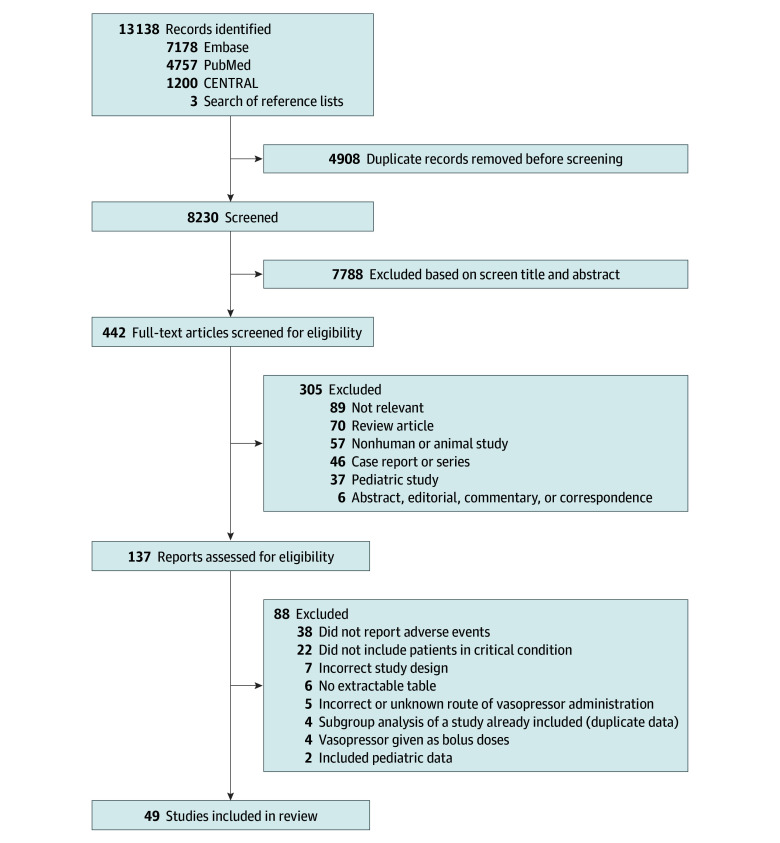
PRISMA Diagram of Study Identification, Screening, Inclusion, and Exclusion From Meta-Analysis CENTRAL indicates Cochrane Central Register of Controlled Trials.

After removing duplicates, our literature search identified 8230 publications: 8227 from PubMed, Embase, and CENTRAL and an additional 3 studies identified through manual searches of the reference lists of included articles. Following detailed evaluation, 49 studies were included in the systematic review and meta-analysis (Figure 1).^9,26,27,28,29,30,31,32,33,34,35,36,37,38,39,40,41,42,43,44,45,46,47,48,49,50,51,52,53,54,55,56,57,58,59,60,61,62,63,64,65,66,67,68,69,70,71,72,73^ The Cohen κ value for study selection was 0.80.

### Study Characteristics

A summary of the 49 included studies is presented in the Table. Detailed characteristics of the included studies are provided in eTable 2 in Supplement 1. All of the eligible studies were published between 1997 and 2025 and included a total of 33 060 catheters used for PIV vasopressor administration. One study (2.0%) only included the proportion of CVC avoidance without reporting AEs associated with PIV use.^26^ Among the included studies, 14 (28.6%) were prospective observational studies,^27,28,29,30,31,32,33,34,35,36,37,38,39,40^ 31 (63.3%) were retrospective observational studies,^9,26,39,41,42,43,44,45,46,47,48,49,50,51,52,53,54,55,56,57,58,59,60,61,62,63,64,65,66,67,68^ and 4 (8.2%) were RCTs.^69,70,71,72^ Regarding the study settings, 4 studies (8.2%) were conducted in an OR,^27,28,42,70^ 9 (18.4%) in an ED,^31,40,41,51,54,61,66,67,71^ 2 (4.1%) in a ward,^35,62^ 2 (4.1%) in an intermediate care unit,^43,68^ 26 (53.1%) in an ICU,^26,29,30,32,36,37,38,39,44,45,48,49,50,52,53,55,56,57,58,59,60,63,64,65,72,73^ and 6 (12.2%) in a mixture of clinical settings, including EDs, ICUs, and wards.^9,33,34,46,47,69^ The JBI critical appraisal checklist was applied to 45 prevalence studies^9,26,27,28,29,30,31,32,33,34,35,36,37,38,39,40,41,42,43,44,45,46,47,48,49,50,51,52,53,54,55,56,57,58,59,60,61,62,63,64,65,66,67,68,73^ (eTable 3 in Supplement 1), and the 4 RCTs^69,70,71,72^ were assessed using the RoB 2 tool (eTable 4 in Supplement 1). Most of the studies (30 [61.2%]) were classified as having unclear or high risk of bias.^9,28,30,31,32,35,39,40,41,43,44,45,46,51,52,53,56,57,59,60,61,62,63,64,65,67,68,69,72,73^

**Table.** Main Characteristics of Included Studies

Source	Country	Study characteristics			Participant age, y	Protocol	Duration	Participants receiving ultrasonography, No.	Participants, No.		AEs associated with PIV vasopressors, No.	
		Design	Period	Setting					With catheters receiving PIV vasopressors^a^	Avoiding CVC	Minor	Major
Andrews et al,^71^ 2017	Zambia	RCT	2012-2013	ED	NR	NR	NR	NR	17	0	0	0
Asher et al,^30^ 2023	Israel	PS	2022	CCU	Mean (SD), 72.0 (12.3)	NR	Mean, 2.5 d	NR	108	NR	7	0
Aykanat et al,^70^ 2022	Australia	RCT	2019-2020	OR	Mean (SD), 67.0 (9.8)	Yes	NR	NR	30	27	3	0
Ballieu et al,^53^ 2021	US	RS	2014-2016	Neurology ICU	Mean, 59.3	Yes	Mean, 14 h 48 min	NR	125	32	9	0
Bima et al,^62^ 2022	Uganda	RS in 2019, PS in 2020	2019-2020	Ward	Median (IQR), 38.0 (35.0-59.0)	Yes	Median (IQR), 15.0 h (12.0-18.0 h)	NR	23	23	0	0
Cape et al,^29^ 2022	US	PS	2018	ICU	Median (IQR), 63 (44.5-81.5)	Yes	Median (range), 2 h 57 min (5 min to 31 h)	NR	84 (92)	31	3	0
Cardenas-Garcia et al,^58^ 2015	US	PS and RS	2012-2014	ICU	Mean (SD), 72 (15)	Yes	Mean (SD), 49 (22) h	734	734	639	19	0
Christensen et al,^27^ 2024	Sweden	PS	2019-2022	OR	Mean (SD), 71.0 (12.0)	Yes	Median (IQR), 175.5 min (105.0-276.0 min)	13	1004	954	23	0
Dansereau et al,^45^ 2024	US	RS	2020-2021	ICU	Mean (SD), 66.7 (15.2)	Yes	Mean, 26.3 h	NR	250 (382)	146	14	0
Datar et al,^56^ 2018	US	RS	2012-2015	Neurology ICU	Mean (SD), 65 (15)	NR	Mean (SD), 19 (18) h	NR	277	220	9	0
Delaney et al,^9^ 2020	Australia and New Zealand	Post hoc analysis of ARISE trial	2008-2014	ED and ICU	Median (IQR), 65.4 (52.4-75.3)	NR	Median (IQR), 1.3 h (0.7-2.5 h)	NR	389	29	0	0
Delgado et al,^57^ 2016	US	RS	2013-2014	Neurology ICU	Median (range), 62 (14-90)	Yes	Median (range), 14.3 h (1.0-54.3 h)	NR	20	NR	1	0
Fabick et al,^48^ 2023	US	RS	2018-2020	ICU	Median (IQR), 64.0 (53.0-72.0)	Yes	Mean (SD), 38.0 (229.8) h	486	2163	840	58	0
Feng et al,^65^ 2021	China	RS	2015-2019	ICU	Mean (SD), 52.9 (18.7)	NR	NR	NR	116	41	6	0
Gandotra et al,^26^ 2023	Canada	RS	2012-2018	ICU	Mean (SD), 58.7 (15.9)	Yes	Median (IQR), 31.0 h (13.0-63.0 h)	NR	1848	981	NR	NR
Gershengorn et al,^39^ 2023	US	PS	2017-2022	ICU	Median (IQR), 68.7 (58.6-75.7)	NR	Median (IQR), 8.0 d (5.0-13.0 d)	NR	287	NR	13	21
Groetzinger et al,^50^ 2022	US	RS	2019-2020	ICU	Median (IQR), 65.0 (56.0-74.0)	Yes	Median (IQR), 8.7 h (4.7-28.0 h)	38	87	44	3	0
Hallengren et al,^43^ 2017	Sweden	RS	2011-2014	IMCU	Median (IQR), 81.0 (73.0-88.0)	NR	Median (IQR), 13.0 h (8.0-30.0 h)	NR	79	78	0	0
Han and Zhou,^63^ 2024	China	RS	2019-2022	Neurology ICU	Median (IQR), 64.0 (55.0-71.0)	Yes	Median (IQR), 79.3 h (18.9-106.6 h)	NR	273	219	5	0
He and Yin,^64^ 2022	China	RS	2019-2020	ICU	Mean (SD), 54.4 (6.7)	NR	NR	NR	640	NR	27	0
Johnson et al,^72^ 1977	US	RCT	1975-1976	ICU	NR	NR	Mean, >48 h	NR	11	NR	1	0
Karlsson et al,^68^ 2024	Sweden	RS	2020-2023	IMCUs	Median (IQR), 73.5 (65.0-80.0)	Yes	Median (IQR), 21.0 h (9.0-38.0 h)	472	472	372	39	7
Kilian et al,^51^ 2022	US	RS	2018-2019	ED	Mean, 64.3	Yes	Mean, 2.3 d	NR	34	4	0	0
Lewis et al,^55^ 2019	US	RS	2015-2016	ICU	Median (IQR), 75.0 (64.0-83.0)	NR	Median (IQR), 11.5 h (NR)	NR	202	109	8	0
Marques et al,^34^ 2022	Rwanda	PS	2019	ED and ICU	Median (IQR), 49.0 (33.0-65.0)	Yes	Median (IQR), 19.0 h (8.5-37.0 h)	NR	64	57	2	0
Marti et al,^52^ 2022	US	RS	2020-2021	ICU	NR	Yes	Mean, 18.7 h	NR	79 (129)	45	3	0
McCurry et al,^66^ 2025	US	RS	2014-2024	ED	Median (IQR), 66.5 (54.3-78.0)	Yes	Median (IQR), 444.0 min (196.0-1314.0 min)	9	80	54	0	0
Medlej et al,^31^ 2018	Lebanon	PS	2013-2015	ED	Mean, 70	NR	Median (IQR), 14.0 h (7.0-40.0 h)	NR	55	42	3	0
Messina et al,^41^ 2021	Italy	RS	2018-2019	ED	Median (IQR), 77.0 (69.0-85.0)	Yes	Median (IQR), 15.0 h (9.0-25.8 h)	NR	127	116	5	0
Munroe et al,^46^ 2024	US	RS	2020-2022	Mixed	Median (IQR), 70.0 (60.0-78.0)	NR	Median (IQR), 2.0 h (2.0-4.0 h)	NR	400	135	0	0
Munroe et al,^67^ 2025	US	RS	2018-2022	ED	Median (IQR), 63.0 (53.0-72.0)	Yes	NR	NR	490	260	3	0
Nguyen et al,^54^ 2021	US	RS	2016-2019	ED	Median (IQR), 60.0 (51.0-70.0)	Yes	Median (IQR), 62.0 min (31.0-142.0 min)	NR	177	29	4	0
Padmanaban et al,^37^ 2020	India	PS	2018-2018	ICU	Mean (SD), 55 (4)	NR	Mean, 10.3 h	NR	122	83	1	0
Pancaro et al,^42^ 2020	The Netherlands	RS	2012-2016	OR	NR	Yes	NR	NR	14 385	NR	5	0
Petros et al,^33^ 2025	Ethiopia	PS	2021	ED and ICU	Median (IQR), 48.5 (35.0-62.0)	NR	Median (IQR), 2.1 d (1.2-2.6 d)	NR	250	NR	3	0
Powell et al,^49^ 2023	US	RS	2021	ICU	Median (IQR), 69.5 (57.0-78.0)	Yes	Median (IQR), 6.0 h (3.3-11.3 h)	NR	98	36	0	0
Prasanna et al,^73^ 2021	US	RS	2016-2019	ICU	Median (IQR), 66.0 (57.0-76.0)	NR	7.8 (9.3) d	248	248	219	2	2
Putland et al,^61^ 2006	Australia	RS	1998-2003	ED	Median (range), 36 (18-55)	Yes	Median (range), 19.5 h (10.0 min to 11.4 d)	NR	220	NR	7	0
Ramanan et al,^69^ 2025	Australia	RCT	2023-2024	ED and ICU	Mean (SD), 68.2 (16.0)	Yes	Mean (SD), 29.4 (77.6) h	NR	40	4	7	0
Ruchti et al,^32^ 2021	Australia	PS	2017-2018	ICU	Mean (SD), 64.95 (15.14)	Yes	Mean (SD), 2.7 (3.5) h	NR	100	80	2	0
Sardaneh et al,^60^ 2021	Australia	RS	2018-2019	Neurology ICU	Median (IQR), 67.0 (56.0-81.0)	NR	Median (IQR), 12.0 (3.0-25.0) h	NR	81	69	0	0
Schmucki et al,^28^ 2025	Switzerland	PS	2020-2021	OR	Median (range), 70 (18-100)	Yes	Median (range), 102 min (0-824 min)	NR	(1561)	NR	252	0
Shyu et al,^44^ 2025	US	RS	2020-2023	ICU	Median (IQR), 68.0 (57.0-77.0)	Yes	Median (IQR), 29.0 h (12.0-67.0 h)	NR	3734	926	30	1
Spiegel et al,^40^ 2020	US	PS	2016-2017	ED	NR	Yes	NR	NR	119	NR	1	0
Stolz et al,^59^ 2022	Australia	RS	2019-2020	ICU	Mean (SD), 65.6 (16.1)	NR	Mean (SD), 31.1 (57.7) h	NR	194	39	72	0
Vitharana et al,^35^ 2023	Sri Lanka	PS	2022	Ward	Range, 15-94	Yes	Median (range), 77.8 h (12-384 h)	NR	52	48	20	0
Yasuda et al,^36^ 2022	Japan	Secondary analysis of previous PS	2018	ICU	NR	NR	NR	NR	(88)	NR	19	0
Yerke et al,^38^ 2024	US	PS	2019-2021	ICU	Median (IQR), 63.0 (55.0-71.0)	Yes	Median (IQR), 5.8 h (2.0-19.7 h)	316	635	311	35	0
Zichichi et al,^47^ 2025	US	RS	2022	Mixed	Mean (SD), 68.0 (16.3)	Yes	Median (IQR), 10.0 h (4.0-21.0 h)	29	198	117	11	0

Abbreviations: AE, adverse event; CCU, cardiovascular care unit; CVC, central venous catheter; ED, emergency department; ICU, intensive care unit; IMCU, intermediate care unit; NR, not reported; OR, operation room; PIV, peripheral intravenous; PS, prospective study; RCT, randomized clinical trial; RS, retrospective study.

^a^ Numbers in parentheses indicate the number of catheters.

### Characteristics of Patients Receiving PIV Vasopressors

The mean or median age of participants in the 49 included studies ranged between 36 and 81 years. All but 5 studies (10.2%) used standard short PIV catheters; 4 (8.2%) used midline catheters^39,40,68,73^ and 1 (2.0%)^67^ used both catheter types. Among the 16 studies (32.7%) that recorded PIV catheter gauge, 57 of 2077 catheters receiving vasopressors (2.7%) had a gauge of 22 G or greater,^29,31,37,47,50,54,65,66^ while 2020 catheters (97.3%) had a gauge less than 22 G.^30,40,51,53,57,68,70,73^ Of the 18 studies (36.7%) reporting PIV catheter location, 2119 of 4103 patients (51.6%) had catheters placed at or above the antecubital fossa,^29,31,33,40,45,47,49,50,51,55,64,66,68,73^ 1898 patients (46.3%) had placement below the antecubital fossa,^29,31,33,37,38,45,47,49,50,51,54,55,64,65,66^ and 86 patients (2.1%) had catheters placed at other sites, such as the neck or leg.^29,31,33,37,45,54,66^ Across included studies evaluating AEs, norepinephrine was the most frequently investigated vasopressor, administered to 23 118 catheters (69.9%) in 30 studies (61.2%).^9,27,28,29,31,32,33,35,36,37,38,41,42,43,45,46,47,48,49,50,51,52,54,55,58,62,64,65,68,70^ Other vasopressors evaluated included epinephrine (11 studies [22.4%], 643 catheters [1.9%]),^9,33,37,45,46,47,48,51,52,55,61^ phenylephrine (11 studies [22.4%], 1756 catheters [5.3%]),^45,46,47,48,51,52,53,55,56,57,58^ dopamine (8 studies [16.3%], 375 catheters [1.1%]),^31,33,37,46,48,55,58,71^ vasopressin (7 studies [14.3%], 440 catheters [1.3%]),^37,45,48,52,55,66,72^ and metaraminol (4 studies [8.2%], 462 catheters [1.4%]).^9,32,60,63^ Detailed AE features are summarized in eTable 5 in Supplement 1.

### Primary Analysis of AE Incidence and CVC Avoidance Proportion

**Figure 2.**
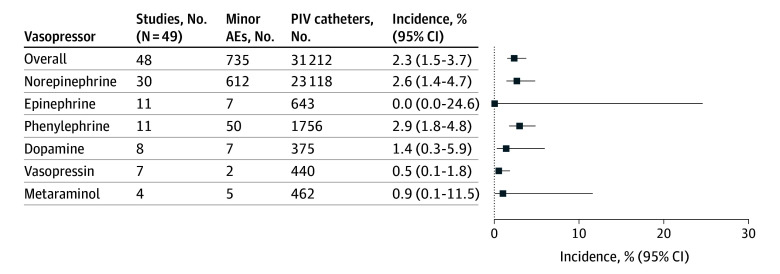
Forest Plot Showing Pooled Incidence of Minor Adverse Events (AEs) Associated With Individual Vasopressor Agents Minor AEs included pain at injection site, local tissue swelling or edema, infiltration, extravasation, tissue cannulation, cellulitis, and thrombophlebitis. PIV indicates peripheral intravenous.

**Figure 3.**
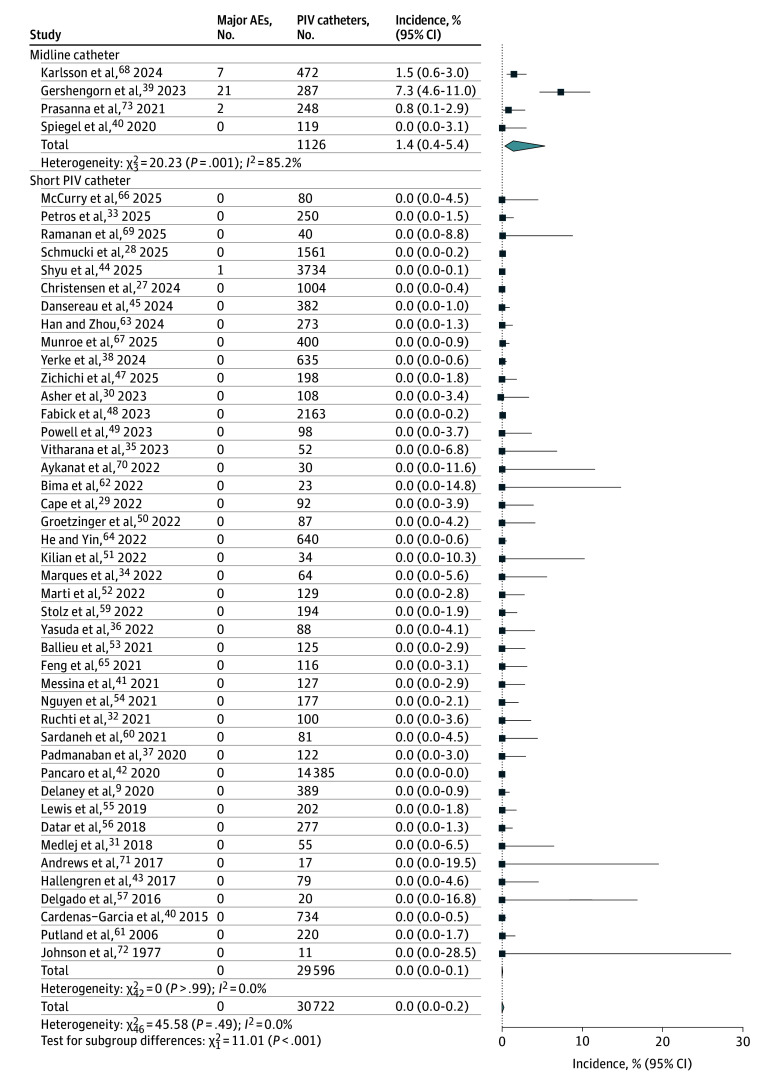
Forest Plot Showing Pooled Incidence of Major Adverse Events (AEs), Stratified by Midline or Short Peripheral Intravenous (PIV) Catheter Use Major AEs included venous thromboembolism and tissue necrosis. Squares indicate incidence rates, with horizontal lines indicating 95% CIs; diamonds indicate pooled estimates, with outer points of the diamonds indicating the 95% CIs.

**Figure 4.**
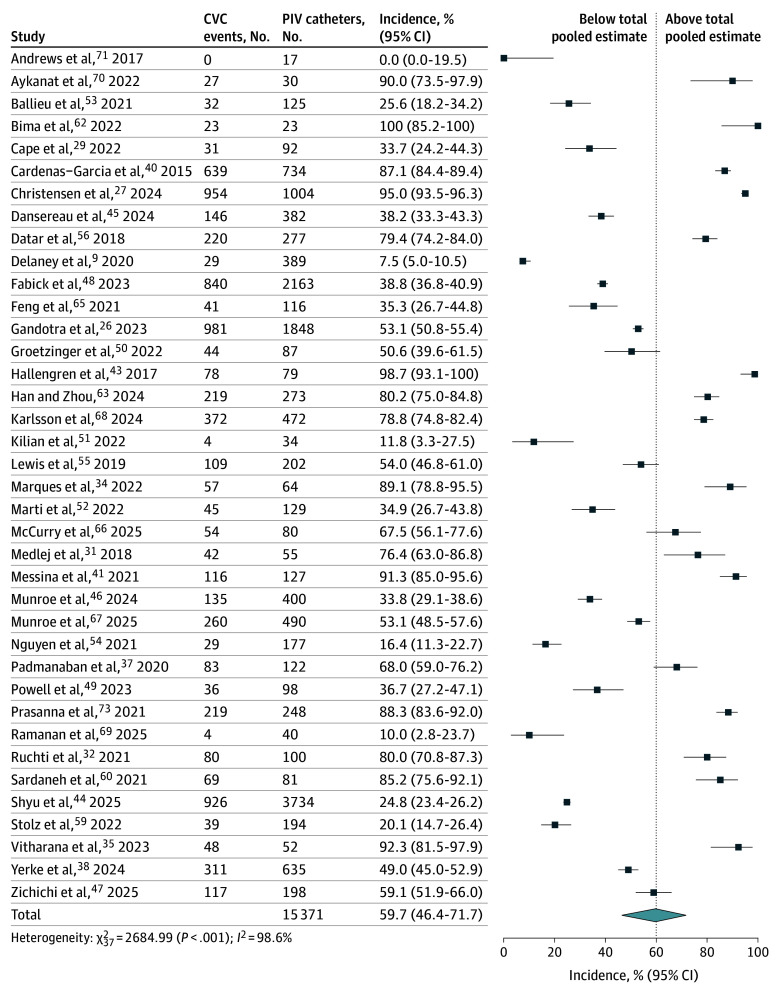
Forest Plot Showing Pooled Incidence of Central Venous Catheter (CVC) Avoidance Squares indicate incidence rates, with horizontal lines indicating 95% CIs; diamond indicates the total pooled estimate, with outer points of the diamond indicating the 95% CI. PIV indicates peripheral intravenous.

Of the 49 included studies, all but 1 (2.0%)^26^ reported AE outcomes. Among 31 212 catheters, 735 minor AEs were observed, most commonly hematoma, extravasation, and skin discoloration. The pooled minor AE incidence was 2.6% (95% CI, 1.4%-4.7%; *I*^2^ = 93.9%) for norepinephrine, 0.0% (95% CI, 0.0%-24.6%; *I*^2^ = 0.0%) for epinephrine, 2.9% (95% CI, 1.8%-4.8%; *I*^2^ = 25.8%) for phenylephrine, 1.4% (95% CI, 0.3%-5.9%; *I*^2^ = 0.0%) for dopamine, 0.5% (95% CI, 0.1%-1.8%; *I*^2^ = 0.0%) for vasopressin, and 0.9% (95% CI, 0.1%-11.5%; *I*^2^ = 0.0%) for metaraminol (Figure 2 and eFigure 1 in Supplement 1). When considering all vasopressors collectively, the pooled minor AE incidence was 2.3% (95% CI, 1.5%-3.7%; *I*^2^ = 95.2%) (eFigure 2 in Supplement 1). Thirty-one major AEs were reported, including 30 VTE events (96.8%) and 1 case of tissue necrosis requiring fasciotomy (3.2%). All 30 VTEs occurred in the 4 studies (8.2%) using midline catheters (1126 total catheters),^39,40,68,73^ with pooled incidence of 1.4% (95% CI, 0.4%-5.4%; *I*^2^ = 85.2%). In contrast, in 43 studies of short PIV catheters that reported AEs (87.8% of total studies, including all but 1 of the studies of short PIV catheters^26^), only 1 major AE (tissue necrosis) was observed across 29 596 total catheters, with pooled incidence of 0.0% (95% CI, 0.0%-0.0%; *I*^2^ = 0.0%). The difference in major AE incidence between the midline and short PIV subgroups was statistically significant (*P* < .001) (Figure 3). One study (2.0%) included both catheter types and could not be stratified.^67^ When considering both catheter types collectively, the pooled incidence of major AEs was 0.0% (95% CI, 0.0%-0.2%; *I*^2^ = 0.0%). In 38 studies (77.6%) including 15 371 catheters, CVC avoidance ranged from 0.0% to 100%, with a pooled proportion of 59.7% (95% CI, 46.4%-71.7%; *I*^2^ = 98.6%) (Figure 4).^9,26,27,29,31,32,34,35,37,38,40,41,43,44,45,46,47,48,49,50,51,52,53,54,55,56,59,60,62,63,65,66,67,68,69,70,71,73^

### Subgroup and Sensitivity Analyses

Significant heterogeneity was observed for AEs and the proportion of CVC avoidance across the included studies, as indicated by high *I*^2^ values. Therefore, we performed subgroup analyses to explore potential sources of heterogeneity. For minor AEs, studies published before 2021 reported significantly lower incidence compared with those published in or after 2021 (1.4% [95% CI, 0.7%-2.8%] vs 3.4% [95% CI, 1.9%-5.9%]; *P* = .048) (eTable 6 in Supplement 1). Retrospective observational studies reported significantly lower incidence (1.5%; 95% CI, 0.8%-2.8%) compared with prospective observational studies (4.5%; 95% CI, 2.4%-8.5%) and RCTs (11.1%; 95% CI, 5.2%-22.3%) (*P* < .001). For proportion of CVC avoidance, studies conducted in the OR and in wards reported a significantly higher proportion of CVC avoidance compared with those conducted in the ICU, ED, or mixed settings. Studies using midline catheters demonstrated a significantly higher proportion of CVC avoidance compared with those using short PIV catheters (83.8% [95% CI, 75.9%-89.4%] vs 58.1% [95% CI, 43.8%-71.2%]; *P* < .001) (eTable 7 in Supplement 1). We conducted 2 sensitivity analyses by excluding studies with a high risk of bias and using the leave-1-out method, which showed that the pooled incidence of minor AEs and CVC avoidance remained consistent (eTables 8 and 9 in Supplement 1).

### Publication Bias

Doi plots and LFK indices demonstrated major asymmetry for minor AEs (LFK, −3.7), major AEs (LFK, −3.5), and CVC avoidance (LFK, 3.0), raising the possibility of publication bias (eFigure 3 in Supplement 1). However, the Peters regression test did not detect significant small-study effects for any outcome. These findings suggest that the asymmetry observed in the Doi plots may be attributable to substantial between-study heterogeneity or unmeasured moderating variables rather than selective reporting.

## Discussion

This study was a comprehensive systematic review and meta-analysis of PIV vasopressor use in critically ill adults, incorporating data from 49 studies and 33 060 catheters. Our results showed that PIV vasopressor use was associated with a low AE incidence. The pooled incidence of minor AEs was 2.3% overall. The pooled incidence of major AEs was 0.0% in studies using short PIV catheters, compared with 1.4% in studies using midline catheters. Additionally, PIV vasopressors enabled avoidance of CVC placement in 59.7% of cases in which PIV catheters were used, although this varied from 0.0% to 100% across individual studies. A recent meta-analysis found that approximately 3% of patients with a CVC placed for 3 days experienced at least 1 serious complication, defined as arterial cannulation, pneumothorax, infection, or deep vein thrombosis.^74^ These observations suggest that with appropriate monitoring, short-term vasopressor administration via short PIV catheters placed in proximal veins may represent a safe alternative to CVCs, particularly when rapid initiation of therapy is required or when central venous access is not immediately available.

### AEs

Prior reviews have reported low complication rates with PIV vasopressors, with extravasation occurring in 2% to 3%.^10,12^ We found that the incidence of minor AEs varied markedly by study design. Retrospective observational studies demonstrated a significantly lower incidence than prospective studies and RCTs, a pattern likely attributable to underascertainment of mild, self-limited events in database-driven cohorts, particularly large administrative studies (eg, Pancaro et al^42^ [14 385 patients]). A similar pattern was observed in temporal analyses: studies published after 2021 reported significantly higher minor AE incidence than earlier studies. This trend coincided with the Surviving Sepsis Campaign recommendation to initiate vasopressors peripherally rather than delaying therapy for central access and is likely attributable to broader adoption and increased reporting vigilance.^22^

Regarding major AEs, the higher VTE incidence observed with midline catheters aligns with broader vascular-access literature indicating a meaningful thrombosis risk with midline catheters, including an estimated rate of 22 per 1000 catheter-days in a recent review.^75,76^ The apparent difference in VTE risk between midline and short PIV catheters may be explained by important confounders, including catheter dwell time, insertion site, and catheter diameter. Prior studies have demonstrated that longer durations of vascular device placement are associated with an increased risk of VTE.^77^ Midline catheters can remain in situ for more than a week, whereas PIV catheters are typically used for only hours to days.^78^ Additionally, short PIV catheters generally have smaller diameters and more distal insertion sites.^79,80^ These results highlight the need for careful patient selection, duration limits, and AE surveillance when midline catheters are used.

### CVC Avoidance

CVC avoidance during vasopressor therapy varied widely, driven by differences in clinical setting, severity of the patient’s condition, protocols, clinician preference, institutional policy, and resource availability. In ED and ICU settings, low avoidance reflected protocols where peripheral administration functions primarily as a temporary bridge to facilitate rapid resuscitation while awaiting standard central access, rather than a replacement for it.^9,51,54^ Conversely, the OR demonstrated high avoidance due to transient anesthesia-related hypotension and intensive monitoring that support short-term peripheral use.^27,70^ High avoidance was also observed when protocols are explicitly designed to spare CVCs through specific safety measures, such as the use of midline catheters^73^ or ultrasonography-guided placement with extended observation periods.^58^ In resource-limited settings where CVC equipment or expertise was unavailable, peripheral administration was default standard of care, resulting in near-total CVC avoidance.^35,62^

### Strengths and Limitations

Compared with previous reviews,^10,11,12,81^ our study offers additional methodologic and clinical insights. First, with 49 studies, it represents the largest meta-analysis on PIV vasopressor use to date, to our knowledge. Second, we conducted stratified analyses by individual vasopressor agents, allowing clearer assessment of safety profiles for each drug. Third, we included studies that examined newer vascular access devices, particularly midline catheters, which are gaining popularity in clinical practice. Fourth, we analyzed the pooled proportion of CVC avoidance, providing a practical outcome measure with direct implications for procedural decision-making and patient safety. Fifth, we performed subgroup and sensitivity analyses to explore heterogeneity and assess the robustness of our results.

Several limitations should also be acknowledged in this study. First, although a thorough and comprehensive literature search was conducted without language restrictions, the possibility of missing relevant studies cannot be completely excluded. Second, the majority of the included studies were retrospective cohort studies, which could inherently carry a risk of reporting bias and potentially underestimate the incidence of AEs. Third, most of the included studies were judged as having unclear or high risk of bias, primarily due to the absence of clear, valid, and standardized methods for assessing AEs and the lack of reported CIs. Fourth, considerable heterogeneity was observed across studies, reflecting variations in study design, clinical protocols, and health care settings. This heterogeneity may affect the generalizability of our findings to broader populations. Fifth, due to limited or inconsistent reporting across studies, we were unable to perform analyses based on the dosage or concentration of vasopressors administered. Sixth, our findings should not be interpreted as evidence to replace CVCs with PIVs for vasopressor administration, as no high-quality RCTs to our knowledge have directly compared these approaches with respect to AE evaluation. Future research should prioritize multicenter RCTs directly comparing PIV and CVC routes using standardized AE outcomes while also evaluating the impact of dose, concentration, infusion duration, and catheter characteristics.

## Conclusions

In this systematic review and meta-analysis of critically ill adult patients, we found that PIV vasopressor administration was associated with a low incidence of AEs and with avoidance of CVC placement in a substantial proportion of patients. The findings support the use of short-term vasopressor administration through short PIV catheters placed in proximal veins under appropriate monitoring.

## Notes

Supplement 1. **eAppendix.** Deviations From Registered Protocol **eTable 1.** Electronic Search Strategies **eTable 2.** Detailed Demographic and Clinical Characteristics of Patients Who Received Peripheral Intravenous Vasopressors **eTable 3.** Quality Assessment of Prevalence Studies Based on the JBI Checklist **eTable 4.** Assessment of Risk of Bias in Randomized Trials Using the Revised Cochrane RoB 2 Tool **eTable 5.** Details of Adverse Event Features **eTable 6.** Summary of Subgroup Analysis of Minor Adverse Events **eTable 7.** Summary of Subgroup Analysis of Central Venous Catheter Avoidance Proportion **eTable 8.** Sensitivity Analyses of the Pooled Incidence Proportion of Minor Adverse Events: Excluding High-Risk-of-Bias Studies and Using the Leave-One-Out Method **eTable 9.** Sensitivity Analyses of the Pooled Proportion of CVC Avoidance: Excluding High-Risk-of-Bias Studies and Using the Leave-1-Out Method **eFigure 1.** Forest Plot Showing the Pooled Incidence Proportions of Minor Adverse Events Associated With Individual Vasopressor Agents **eFigure 2.** Forest Plot of Pooled Incidence Proportion of Minor Adverse Events Across All Vasopressor Agents **eFigure 3.** Doi Plot Visualization and the Luis Furuya-Kanamori (LFK) Asymmetry Index for Publication Bias Assessment
eReferences

Supplement 2.
Data Sharing Statement

## References

[1] Vincent JL, De Backer D. Circulatory shock. N Engl J Med. 2013;369(18):1726-1734. doi:10.1056/NEJMra1208943 24171518

[2] Black LP, Puskarich MA, Smotherman C, Miller T, Fernandez R, Guirgis FW. Time to vasopressor initiation and organ failure progression in early septic shock. J Am Coll Emerg Physicians Open. 2020;1(3):222-230. doi:10.1002/emp2.12060 33000037 PMC7493499

[3] Colon Hidalgo D, Patel J, Masic D, Park D, Rech MA. Delayed vasopressor initiation is associated with increased mortality in patients with septic shock. J Crit Care. 2020;55:145-148. doi:10.1016/j.jcrc.2019.11.004 31731173

[4] Humphreys J, Johnston JH, Richardson JC. Skin necrosis following intravenous noradrenaline. BMJ. 1955;2(4950):1250-1252. doi:10.1136/bmj.2.4950.1250 13269844 PMC1981272

[5] Kahn JM, Kress JP, Hall JB. Skin necrosis after extravasation of low-dose vasopressin administered for septic shock. Crit Care Med. 2002;30(8):1899-1901. doi:10.1097/00003246-200208000-00038 12163813

[6] Ramadan H, Metin Aksu N, Akkas M, Husamettin Akkucuk M, Coskun F, Cetinkaya Sardan Y. Mechanical and infectious complications developing due to central venous catheterizations in the emergency department. Med Glas (Zenica). 2013;10(1):40-45. 23348159

[7] Patel AR, Patel AR, Singh S, Singh S, Khawaja I. Central line catheters and associated complications: a review. Cureus. 2019;11(5):e4717. doi:10.7759/cureus.4717 31355077 PMC6650175

[8] Woodfall K, van Zundert A. Central venous access: an update on modern techniques to avoid complications. Healthcare (Basel). 2025;13(10):1168. doi:10.3390/healthcare13101168 40428004 PMC12111573

[9] Delaney A, Finnis M, Bellomo R, . Initiation of vasopressor infusions via peripheral versus central access in patients with early septic shock: a retrospective cohort study. Emerg Med Australas. 2020;32(2):210-219. doi:10.1111/1742-6723.13394 31599084

[10] Owen VS, Rosgen BK, Cherak SJ, . Adverse events associated with administration of vasopressor medications through a peripheral intravenous catheter: a systematic review and meta-analysis. Crit Care. 2021;25(1):146. doi:10.1186/s13054-021-03553-1 33863361 PMC8050944

[11] Tran QK, Mester G, Bzhilyanskaya V, . Complication of vasopressor infusion through peripheral venous catheter: a systematic review and meta-analysis. Am J Emerg Med. 2020;38(11):2434-2443. doi:10.1016/j.ajem.2020.09.047 33039229

[12] Tian DH, Smyth C, Keijzers G, . Safety of peripheral administration of vasopressor medications: a systematic review. Emerg Med Australas. 2020;32(2):220-227. doi:10.1111/1742-6723.13406 31698544

[13] McInnes MDF, Moher D, Thombs BD, ; and the PRISMA-DTA Group. Preferred reporting items for a systematic review and meta-analysis of diagnostic test accuracy studies: the PRISMA-DTA statement. JAMA. 2018;319(4):388-396. doi:10.1001/jama.2017.19163 29362800

[14] Ricard JD, Salomon L, Boyer A, . Central or peripheral catheters for initial venous access of ICU patients: a randomized controlled trial. Crit Care Med. 2013;41(9):2108-2115. doi:10.1097/CCM.0b013e31828a42c5 23782969

[15] Loubani OM, Green RS. A systematic review of extravasation and local tissue injury from administration of vasopressors through peripheral intravenous catheters and central venous catheters. J Crit Care. 2015;30(3):653.e9-653.e17. doi:10.1016/j.jcrc.2015.01.014 25669592

[16] Adams DZ, Little A, Vinsant C, Khandelwal S. The midline catheter: a clinical review. J Emerg Med. 2016;51(3):252-258. doi:10.1016/j.jemermed.2016.05.029 27397766

[17] Alexandrou E, Ramjan LM, Spencer T, . The use of midline catheters in the adult acute care setting—clinical implications and recommendations for practice. Journal of the Association for Vascular Access. 2011;16(1):35-41. doi:10.2309/java.16-1-5

[18] Munn Z, Moola S, Lisy K, Riitano D, Tufanaru C. Systematic reviews of prevalence and incidence. In: *JBI Manual for Evidence Synthesis*. Joanna Briggs Institute; 2017. Accessed February 5, 2026. https://jbi-global-wiki.refined.site/space/MANUAL/355863557

[19] Sterne JAC, Savović J, Page MJ, . RoB 2: a revised tool for assessing risk of bias in randomised trials. BMJ. 2019;366:l4898. doi:10.1136/bmj.l4898 31462531

[20] Lin L, Chu H. Meta-analysis of proportions using generalized linear mixed models. Epidemiology. 2020;31(5):713-717. doi:10.1097/EDE.0000000000001232 32657954 PMC7398826

[21] Higgins JPT, Thompson SG, Deeks JJ, Altman DG. Measuring inconsistency in meta-analyses. BMJ. 2003;327(7414):557-560. doi:10.1136/bmj.327.7414.557 12958120 PMC192859

[22] Evans L, Rhodes A, Alhazzani W, . Surviving Sepsis Campaign: international guidelines for management of sepsis and septic shock 2021. Intensive Care Med. 2021;47(11):1181-1247. doi:10.1007/s00134-021-06506-y 34599691 PMC8486643

[23] Furuya-Kanamori L, Barendregt JJ, Doi SAR. A new improved graphical and quantitative method for detecting bias in meta-analysis. Int J Evid Based Healthc. 2018;16(4):195-203. doi:10.1097/XEB.0000000000000141 29621038

[24] Peters JL, Sutton AJ, Jones DR, Abrams KR, Rushton L. Comparison of two methods to detect publication bias in meta-analysis. JAMA. 2006;295(6):676-680. doi:10.1001/jama.295.6.676 16467236

[25] Borges Migliavaca C, Stein C, Colpani V, Barker TH, Munn Z, Falavigna M; Prevalence Estimates Reviews–Systematic Review Methodology Group (PERSyst). How are systematic reviews of prevalence conducted? a methodological study. BMC Med Res Methodol. 2020;20(1):96. doi:10.1186/s12874-020-00975-3 32336279 PMC7184711

[26] Gandotra S, Wunsch H, Bosch NA, Walkey AJ, Teja B. Reducing central venous catheter use through adoption of guidelines for peripheral catheter-based vasopressor delivery. Ann Am Thorac Soc. 2023;20(8):1219-1223. doi:10.1513/AnnalsATS.202212-1060RL 37220220

[27] Christensen J, Andersson E, Sjöberg F, . Adverse events of peripherally administered norepinephrine during surgery: a prospective multicenter study. Anesth Analg. 2024;138(6):1242-1248. doi:10.1213/ANE.0000000000006806 38180886

[28] Schmucki R, Rüst CA, Filipovic M. Intra-operative norepinephrine via peripheral venous catheter is safe: a short scientific report. Eur J Anaesthesiol. 2025;42(2):172-173. doi:10.1097/EJA.0000000000002080 39744746

[29] Cape KM, Jones LG, Weber ML, Elefritz JL. Implementation of a protocol for peripheral intravenous norepinephrine: does it save central line insertion, is it safe? J Pharm Pract. 2022;35(3):347-351. doi:10.1177/0897190020977712 33267711

[30] Asher E, Karameh H, Nassar H, ; Jerusalem Platelets Thrombosis and Intervention in Cardiology (JUPITER-16) Study Group. Safety and outcomes of peripherally administered vasopressor infusion in patients admitted with shock to an intensive cardiac care unit—a single-center prospective study. J Clin Med. 2023;12(17):5734. doi:10.3390/jcm12175734 37685801 PMC10488618

[31] Medlej K, Kazzi AA, El Hajj Chehade A, . Complications from administration of vasopressors through peripheral venous catheters: an observational study. J Emerg Med. 2018;54(1):47-53. doi:10.1016/j.jemermed.2017.09.007 29110979

[32] Ruchti VE, Wibrow BA, Seet J, Jacques A, Jha N, Anstey MH. A prospective comparison of peripheral metaraminol versus dilute noradrenaline in the intensive care unit. Anaesth Intensive Care. 2021;49(2):144-146. doi:10.1177/0310057X20984794 33853391

[33] Petros A, Melkie A, Kotiso KS, . Peripheral line for vasopressor administration: prospective multicenter observational cohort study for survival and safety. PLoS One. 2025;20(10):e0333275. doi:10.1371/journal.pone.0333275 41082535 PMC12517475

[34] Marques CG, Mwemerashyaka L, Martin K, . Utilisation of peripheral vasopressor medications and extravasation events among critically ill patients in Rwanda: a prospective cohort study. Afr J Emerg Med. 2022;12(2):154-159. doi:10.1016/j.afjem.2022.03.006 35505668 PMC9046616

[35] Vitharana HS, Amarasena R. Study on ward-based practice of vasopressor administration for patients with sepsis, in National Hospital of Sri Lanka (NHSL). Sri Lankan Journal of Anaesthesiology. 2023;31(2):130-135. doi:10.4038/slja.v31i2.9144

[36] Yasuda H, Rickard CM, Marsh N, ; AMOR-NUS study group. Risk factors for peripheral intravascular catheter-related phlebitis in critically ill patients: analysis of 3429 catheters from 23 Japanese intensive care units. Ann Intensive Care. 2022;12(1):33. doi:10.1186/s13613-022-01009-5 35394571 PMC8994002

[37] Padmanaban A, Venkataraman R, Rajagopal S, Devaprasad D, Ramakrishnan N. Feasibility and safety of peripheral intravenous administration of vasopressor agents in resource-limited settings. J Crit Care Med (Targu Mures). 2020;6(4):210-216. doi:10.2478/jccm-2020-0030 33200091 PMC7648440

[38] Yerke JR, Mireles-Cabodevila E, Chen AY, . Peripheral administration of norepinephrine: a prospective observational study. Chest. 2024;165(2):348-355. doi:10.1016/j.chest.2023.08.019 37611862 PMC10851275

[39] Gershengorn HB, Basu T, Horowitz JK, . The association of vasopressor administration through a midline catheter with catheter-related complications. Ann Am Thorac Soc. 2023;20(7):1003-1011. doi:10.1513/AnnalsATS.202209-814OC 37166852 PMC12335012

[40] Spiegel RJ, Eraso D, Leibner E, Thode H, Morley EJ, Weingart S. The utility of midline intravenous catheters in critically ill emergency department patients. Ann Emerg Med. 2020;75(4):538-545. doi:10.1016/j.annemergmed.2019.09.018 31882244

[41] Messina A, Milani A, Morenghi E, . Norepinephrine infusion in the emergency department in septic shock patients: a retrospective 2-years safety report and outcome analysis. Int J Environ Res Public Health. 2021;18(2):824. doi:10.3390/ijerph18020824 33478004 PMC7835753

[42] Pancaro C, Shah N, Pasma W, . Risk of major complications after perioperative norepinephrine infusion through peripheral intravenous lines in a multicenter study. Anesth Analg. 2020;131(4):1060-1065. doi:10.1213/ANE.0000000000004445 32925324

[43] Hallengren M, Åstrand P, Eksborg S, Barle H, Frostell C. Septic shock and the use of norepinephrine in an intermediate care unit: mortality and adverse events. PLoS One. 2017;12(8):e0183073. doi:10.1371/journal.pone.0183073 28837628 PMC5570296

[44] Shyu D, Ingraham NE, Linke CA, . Overview of peripheral vasopressor use in an academic health system. Ann Am Thorac Soc. 2025;22(8):1201-1209. doi:10.1513/AnnalsATS.202411-1135OC 40126143 PMC12329328

[45] Dansereau AC, Marti KE, Mah JW, Pugliese NM. Evaluation of the safety and efficacy of peripheral vasopressors to decrease central line placement and associated bloodstream infections. J Infect Prev. 2024;25(5):153-160. doi:10.1177/17571774241245437 39493308 PMC11528565

[46] Munroe ES, Heath ME, Eteer M, . Use and outcomes of peripheral vasopressors in early sepsis-induced hypotension across Michigan hospitals: a retrospective cohort study. Chest. 2024;165(4):847-857. doi:10.1016/j.chest.2023.10.027 37898185 PMC11214906

[47] Zichichi A, Wallace R, Daniell J, Rouse G, Ahearn P, Ammar M. Safety of peripherally infused sympathomimetic vasopressors in the intensive care unit and emergency department. Ann Pharmacother. 2025;59(5):397-405. doi:10.1177/10600280241284796 39411928

[48] Fabick AC, Hawn JM, Barwick KW, Weeda ER, Goodwin AJ, Bell CM. Comparison of extravasation events related to the peripheral administration of vasopressors prior to and following implementation of an institutional protocol. J Am Coll Clin Pharm. 2023;6(7):709-717. doi:10.1002/jac5.1844

[49] Powell SM, Faust AC, George S, Townsend R, Eubank D, Kim R. Effect of peripherally infused norepinephrine on reducing central venous catheter utilization. J Infus Nurs. 2023;46(4):210-216. doi:10.1097/NAN.0000000000000508 37406335

[50] Groetzinger LM, Williams J, Svec S, Donahoe MP, Lamberty PE, Barbash IJ. Peripherally infused norepinephrine to avoid central venous catheter placement in a medical intensive care unit: a pilot study. Ann Pharmacother. 2022;56(7):773-781. doi:10.1177/10600280211053318 34674566

[51] Kilian S, Surrey A, McCarron W, Mueller K, Wessman BT. Vasopressor administration via peripheral intravenous access for emergency department stabilization in septic shock patients. Indian J Crit Care Med. 2022;26(7):811-815. doi:10.5005/jp-journals-10071-24243 36864853 PMC9973174

[52] Marti K, Hartley C, Sweeney E, Mah J, Pugliese N. Evaluation of the safety of a novel peripheral vasopressor pilot program and the impact on central line placement in medical and surgical intensive care units. Am J Health Syst Pharm. 2022;79(suppl 3):S79-S85. doi:10.1093/ajhp/zxac144 35605137 PMC9384090

[53] Ballieu P, Besharatian Y, Ansari S. Safety and feasibility of phenylephrine administration through a peripheral intravenous catheter in a neurocritical care unit. J Intensive Care Med. 2021;36(1):101-106. doi:10.1177/0885066619887111 31757173

[54] Nguyen TT, Surrey A, Barmaan B, . Utilization and extravasation of peripheral norepinephrine in the emergency department. Am J Emerg Med. 2021;39:55-59. doi:10.1016/j.ajem.2020.01.014 31959524

[55] Lewis T, Merchan C, Altshuler D, Papadopoulos J. Safety of the peripheral administration of vasopressor agents. J Intensive Care Med. 2019;34(1):26-33. doi:10.1177/0885066616686035 28073314

[56] Datar S, Gutierrez E, Schertz A, Vachharajani V. Safety of phenylephrine infusion through peripheral intravenous catheter in the neurological intensive care unit. J Intensive Care Med. 2018;33(10):589-592. doi:10.1177/0885066617712214 28569131

[57] Delgado T, Wolfe B, Davis G, Ansari S. Safety of peripheral administration of phenylephrine in a neurologic intensive care unit: a pilot study. J Crit Care. 2016;34:107-110. doi:10.1016/j.jcrc.2016.04.004 27288620

[58] Cardenas-Garcia J, Schaub KF, Belchikov YG, Narasimhan M, Koenig SJ, Mayo PH. Safety of peripheral intravenous administration of vasoactive medication. J Hosp Med. 2015;10(9):581-585. doi:10.1002/jhm.2394 26014852

[59] Stolz A, Efendy R, Apte Y, Craswell A, Lin F, Ramanan M. Safety and efficacy of peripheral versus centrally administered vasopressor infusion: a single-centre retrospective observational study. Aust Crit Care. 2022;35(5):506-511. doi:10.1016/j.aucc.2021.08.005 34600834

[60] Sardaneh AA, Penm J, Oliver M, Gattas D, McLachlan AJ, Patanwala AE. Pharmacoepidemiology of metaraminol in critically ill patients with shock in a tertiary care hospital. Aust Crit Care. 2021;34(6):573-579. doi:10.1016/j.aucc.2021.01.002 33663948

[61] Putland M, Kerr D, Kelly AM. Adverse events associated with the use of intravenous epinephrine in emergency department patients presenting with severe asthma. Ann Emerg Med. 2006;47(6):559-563. doi:10.1016/j.annemergmed.2006.01.022 16713785

[62] Bima P, Orlotti C, Smart OG, . Norepinephrine may improve survival of septic shock patients in a low-resource setting: a proof-of-concept study on feasibility and efficacy outside the intensive care unit. Pathog Glob Health. 2022;116(6):389-394. doi:10.1080/20477724.2022.2038051 35138990 PMC9387336

[63] Han P, Zhou Y. Safety and efficacy of peripheral metaraminol infusion in patients with neurological conditions: a single-center retrospective observational study. Front Neurol. 2024;15:1398827. doi:10.3389/fneur.2024.1398827 38887388 PMC11180898

[64] He LWD, Yin L. Risk factor analysis and early-warning management of safety in peripheral intravenous norepinephrine infusion for septic shock patients. Chinese General Practice Nursing. 2022;20(10):1418-1421. doi:10.12104/j.issn.1674-4748.2022.10.035

[65] Feng F, Yang W, Zhang Z, Mu C, Li M, Chen Y. Safety of administration of norepinephrine through peripheral vein line in patients with septic shock. Article in Chinese. Zhonghua Wei Zhong Bing Ji Jiu Yi Xue. 2021;33(3):276-280. doi:10.3760/cma.j.cn121430-20200716-00528 33834967

[66] McCurry K, DeWitt K, Upchurch CP, Wren RN. Peripherally administered vasopressin initiated in the emergency department. J Crit Care. 2025;92:155363. doi:10.1016/j.jcrc.2025.155363 41308502

[67] Munroe ES, Co IN, Douglas I, ; NHLBI PETAL Network. Peripheral vasopressor use in early sepsis-induced hypotension. JAMA Netw Open. 2025;8(8):e2529148. doi:10.1001/jamanetworkopen.2025.29148 40864467 PMC12391982

[68] Karlsson H, Afrasiabi A, Ohlsson M, Månsson V, Hartman H, Torisson G. Treating shock with norepinephrine administered in midline catheters in an intermediary care unit: a retrospective cohort study. BMJ Open. 2024;14(12):e091311. doi:10.1136/bmjopen-2024-091311 39806688 PMC11683991

[69] Ramanan M, Apte Y, Watts S, . A randomised, controlled, feasibility trial comparing vasopressors infused via peripheral cannula versus central venous access for critically ill adults: the VIPCA trial. Crit Care Resusc. 2025;27(2):100106. doi:10.1016/j.ccrj.2025.100106 40933726 PMC12417211

[70] Aykanat VM, Myles PS, Weinberg L, Burrell A, Bellomo R. Low-concentration norepinephrine infusion for major surgery: a safety and feasibility pilot randomized controlled trial. Anesth Analg. 2022;134(2):410-418. doi:10.1213/ANE.0000000000005811 34872102

[71] Andrews B, Semler MW, Muchemwa L, . Effect of an early resuscitation protocol on in-hospital mortality among adults with sepsis and hypotension: a randomized clinical trial. JAMA. 2017;318(13):1233-1240. doi:10.1001/jama.2017.10913 28973227 PMC5710318

[72] Johnson WC, Widrich WC, Ansell JE, Robbins AH, Nabseth DC. Control of bleeding varices by vasopressin: a prospective randomized study. Ann Surg. 1977;186(3):369-376. doi:10.1097/00000658-197709000-00015 302111 PMC1396353

[73] Prasanna N, Yamane D, Haridasa N, Davison D, Sparks A, Hawkins K. Safety and efficacy of vasopressor administration through midline catheters. J Crit Care. 2021;61:1-4. doi:10.1016/j.jcrc.2020.09.024 33049486

[74] Teja B, Bosch NA, Diep C, . Complication rates of central venous catheters: a systematic review and meta-analysis. JAMA Intern Med. 2024;184(5):474-482. doi:10.1001/jamainternmed.2023.8232 38436976 PMC12285596

[75] Fabiani A, Aversana N, Santoro M, Sanson G. Complications associated to midline- and long peripheral catheters in adults: systematic review of literature and proposal for a standardized model for data collection. Thromb Res. 2024;236:117-126. doi:10.1016/j.thromres.2024.02.022 38422981

[76] Wen J, Xiong S, Tu Z, . Which is the safer option for adult patients between peripherally inserted central catheters and midline catheters: a meta-analysis. Infect Control Hosp Epidemiol. 2024;46(1):1-8. doi:10.1017/ice.2024.190 39533820

[77] Citla Sridhar D, Abou-Ismail MY, Ahuja SP. Central venous catheter-related thrombosis in children and adults. Thromb Res. 2020;187:103-112. doi:10.1016/j.thromres.2020.01.017 31981840

[78] Moureau N, Sigl G, Hill M. How to establish an effective midline program: a case study of 2 hospitals. Journal of the Association for Vascular Access. 2015;20(3):179-188. doi:10.1016/j.java.2015.05.001

[79] Heng SY, Yap RTJ, Tie J, McGrouther DA. Peripheral vein thrombophlebitis in the upper extremity: a systematic review of a frequent and important problem. Am J Med. 2020;133(4):473-484.e3. doi:10.1016/j.amjmed.2019.08.054 31606488

[80] Geerts W. Central venous catheter-related thrombosis. Hematology Am Soc Hematol Educ Program. 2014;2014(1):306-311. doi:10.1182/asheducation-2014.1.306 25696870

[81] Fernández-Ginés FD, Gómez Sánchez MT, Sánchez Valera M, Tauste Hernández B, Garrido Ortiz M, Cortiñas-Sáenz M. Safe administration of noradrenaline by the peripheral route: a systematic review. Article in Spanish. Farm Hosp. 2025;49(1):T46-T52. doi:10.1016/j.farma.2024.07.004 39079823

