# Supplementary material for: Incidence of Adverse Events in Peripheral Intravenous Vasopressor Use: A Systematic Review and Meta-Analysis
Source: JAMA Netw Open. 2026 Mar 16;9(3):e260710. doi: 10.1001/jamanetworkopen.2026.0710 (PMC12993702; doi:10.1001/jamanetworkopen.2026.0710)
Supplement: Supplement 1. — eAppendix. Deviations From Registered Protocol eTable 1. Electronic Search Strategies eTable 2. Detailed Demographic and Clinical Characteristics of Patients Who Received Peripheral Intravenous Vasopressors eTable 3. Quality Assessment of Prevalence Studies Based on the JBI Checklist eTable 4. Assessment of Risk of Bias in Randomized Trials Using the Revised Cochrane RoB 2 Tool eTable 5. Details of Adverse Event Features eTable 6. Summary of Subgroup Analysis of Minor Adverse Events eTable 7. Summary of Subgroup Analysis of Central Venous Catheter Avoidance Proportion eTable 8. Sensitivity Analyses of the Pooled Incidence Proportion of Minor Adverse Events: Excluding High-Risk-of-Bias Studies and Using the Leave-One-Out Method eTable 9. Sensitivity Analyses of the Pooled Proportion of CVC Avoidance: Excluding High-Risk-of-Bias Studies and Using the Leave-1-Out Method eFigure 1. Forest Plot Showing the Pooled Incidence Proportions of Minor Adverse Events Associated With Individual Vasopressor Agents eFigure 2. Forest Plot of Pooled Incidence Proportion of Minor Adverse Events Across All Vasopressor Agents eFigure 3. Doi Plot Visualization and the Luis Furuya-Kanamori (LFK) Asymmetry Index for Publication Bias Assessment eReferences [file jamanetwopen-e260710-s001.pdf]

Supplementary Online Content

ZhangJian SJ, Niu KY, Chen CB, Seak CJ, Yen CC. Incidence of adverse events in peripheral intravenous vasopressor use: a systematic review and meta-analysis. *JAMA Netw Open*. 2026;9(3):e260710. doi:10.1001/jamanetworkopen.2026.0710

eAppendix. Deviations From Registered Protocol
eTable 1. Electronic Search Strategies
eTable 2. Detailed Demographic and Clinical Characteristics of Patients Who Received Peripheral Intravenous Vasopressors
eTable 3. Quality Assessment of Prevalence Studies Based on the JBI Checklist
eTable 4. Assessment of Risk of Bias in Randomized Trials Using the Revised Cochrane RoB 2 Tool
eTable 5. Details of Adverse Event Features
eTable 6. Summary of Subgroup Analysis of Minor Adverse Events
eTable 7. Summary of Subgroup Analysis of Central Venous Catheter Avoidance Proportion
eTable 8. Sensitivity Analyses of the Pooled Incidence Proportion of Minor Adverse Events: Excluding High-Risk-of-Bias Studies and Using the Leave-One-Out Method
eTable 9. Sensitivity Analyses of the Pooled Proportion of CVC Avoidance: Excluding High-Risk-of-Bias Studies and Using the Leave-1-Out Method
eFigure 1. Forest Plot Showing the Pooled Incidence Proportions of Minor Adverse Events Associated With Individual Vasopressor Agents
eFigure 2. Forest Plot of Pooled Incidence Proportion of Minor Adverse Events Across All Vasopressor Agents
eFigure 3. Doi Plot Visualization and the Luis Furuya-Kanamori (LFK) Asymmetry Index for Publication Bias Assessment
eReferences

This supplementary material has been provided by the authors to give readers additional information about their work.

© 2026 ZhangJian SJ et al. *JAMA Network Open*.

eAppendix. Deviations from registered protocol

The PROSPERO record initially specified that a GRADE assessment of the certainty of evidence would be performed. However, formal methods for applying the GRADE approach to systematic reviews of prevalence outcomes, such as pooled proportions, have not been fully developed or validated in the methodological literature. As a result, we did not perform a GRADE assessment in this review.

The initial literature search for this systematic review and meta-analysis was conducted from database inception through February 28, 2025. During the revision process, we updated the literature search through December 13, 2025, prompted by the emergence of several relevant studies published during the submission and peer-review period.

© 2026 ZhangJian SJ et al. *JAMA Network Open*.

eTable 1. Electronic search strategies

(A) PubMed

Date limits: from inception to December 13, 2025

	Search strategy	Results
1	(((((((((((Norepinephrine[Title/Abstract]) OR (Noradrenaline[Title/Abstract])) OR (Vasopressin[Title/Abstract])) OR (Epinephrine[Title/Abstract])) OR (Adrenaline[Title/Abstract])) OR (Dopamine[Title/Abstract])) OR (Phenylephrine[Title/Abstract])) OR (Metaraminol[Title/Abstract])) OR (Aramine[Title/Abstract])) OR (Vasoconstrictor[Title/Abstract])) OR (Vasoactive[Title/Abstract])) OR (Vasopressor[Title/Abstract])) OR (Sympathomimetic[Title/Abstract])	371,494
2	((((Catheter[Title/Abstract]) OR (Catheters[Title/Abstract])) OR (Intravenous[Title/Abstract])) OR (Administration[Title/Abstract])) OR (Infusions[Title/Abstract])	1,520,855
3	(Peripheral[Title/Abstract]) OR (Midline[Title/Abstract])	740,227
4	2 AND 3	65,621
5	1 AND 4	4,757

(B) Embase

Date limits: from inception to December 13, 2025

	Search strategy	Results
1	norepinephrine:ti,ab,kw OR noradrenaline:ti,ab,kw OR vasopressin:ti,ab,kw OR epinephrine:ti,ab,kw OR adrenaline:ti,ab,kw OR dopamine:ti,ab,kw OR	486,843

© 2026 ZhangJian SJ et al. *JAMA Network Open*.

	phenylephrine:ti,ab,kw OR metaraminol:ti,ab,kw OR aramine:ti,ab,kw OR vasoconstrictor:ti,ab,kw OR vasoactive:ti,ab,kw OR vasopressor:ti,ab,kw OR sympathomimetic:ti,ab,kw	
2	catheter:ti,ab,kw OR catheters:ti,ab,kw OR intravenous:ti,ab,kw OR administration:ti,ab,kw OR infusions:ti,ab,kw	2,181,846
3	peripheral:ti,ab,kw OR midline:ti,ab,kw	1,087,439
4	2 AND 3	107,637
5	1 AND 4	7,178

(C) CENTRAL

Date limits: from inception to December 13, 2025

	Search strategy	Results
1	("norepinephrine"):ti,ab,kw OR (noradrenaline):ti,ab,kw OR (vasopressin):ti,ab,kw OR (epinephrine):ti,ab,kw OR (adrenaline):ti,ab,kw	21,786
2	(dopamine):ti,ab,kw OR (phenylephrine):ti,ab,kw OR (metaraminol):ti,ab,kw OR (aramine):ti,ab,kw	12,259
3	(vasoconstrictor):ti,ab,kw OR (vasoactive):ti,ab,kw OR (vasopressor):ti,ab,kw OR (sympathomimetic):ti,ab,kw	9,460
4	#1 OR #2 OR #3	38,447
5	(catheters):ti,ab,kw OR (intravenous):ti,ab,kw OR (administration):ti,ab,kw OR (infusions):ti,ab,kw	488,535
6	(peripheral):ti,ab,kw OR (midline):ti,ab,kw	66,676
7	#5 AND #6	20,853
8	#4 AND #7	1,200

© 2026 ZhangJian SJ et al. *JAMA Network Open*.

eTable 2. Detailed demographic and clinical characteristics of patients who received peripheral intravenous vasopressors.

Author, year	Male (%)	BMI[a]	Inclusion criteria	Exclusion criteria	Illness severity[a]	Reason for vasopressor	Vasopressor medication, No. (%)	Vasopressor dose[a]	Gauge, No. (%)	Location, No. (%)
Andrews et al, [1] 2017	NR	NR	Patients aged 18 yr or older with septic shock	Patients with hypoxemia and tachypnea; acute decompensate heart failure; gastrointestinal bleeding; incarceration; end stage renal disease	NR	Septic shock	Dopamine: 17 (100)	≧10 mcg/kg/min	NR	NR
Asher et al, [2] 2023	70 (65)	27	Adult patients with hemodynamic shock requiring vasopressor administration	Patients <18yr and pregnant women	NR	Various types of shock	Norepinephrine; Dopamine; Phenylephrine; Vasopressin; Epinephrine	NR	20G: 108 (100)	Above wrist: 108 (100)
Aykanat et al, [3] 2022	10 (30)	NR	Adult patients undergoing major noncardiac surgery and at risk of postoperative complications	Severe hypertension, minor surgery, pregnancy, MAOI use	NA	At risk of hypotension	Norepinephrine: 30 (100)	Maximum rate: 0.1 mcg/kg/min	14G to 18G	Forearm or upper arm (avoiding cubital fossa and wrist)
Ballieu et al, [4] 2021	52 (41.6)	NR	NR	NR	NR	For hemodynamic support and BP	Phenylephrine: 125 (100)	Peak infusion rate: 3.06 mcg/kg/min	18G or larger	In the upper extremity; proximal

© 2026 ZhangJian SJ et al. *JAMA Network Open*.

Author, year	Male (%)	BMI[a]	Inclusion criteria	Exclusion criteria	Illness severity[a]	Reason for vasopressor	Vasopressor medication, No. (%)	Vasopressor dose[a]	Gauge, No. (%)	Location, No. (%)
						augmentation				to the wrist
Bima et al, [5] 2022	12 (52.2)	NR	Adult patients with septic shock requiring vasopressors	NR	qSOFA: 3 (2-3), MEWS: 8 (7-9), NEWS: 12 (8-14)	Septic shock	Norepinephrine: 23 (100)	Median rate: 0.08 mcg/kg/min (0.05-0.10)	Usually 18G	NR
Cape et al, [6] 2022	58 (63)	NR	Patients receiving norepinephrine via PIV for <24hr	NR	NR	Various types of shock	Norepinephrine: 92 (100)	Maximum infusion rate, No. (%) < 0.2 mcg/kg/min: 67 (72.8); 0.21-0.49 mcg/kg/min: 18 (19.6); 0.5-1.0 mcg/kg/min: 4 (4.3); >1.0 mcg/kg/min: 3 (3.3)	16G: 1 (1.1); 18G: 28 (30.4); 20G: 55 (59.8); 22G: 8 (8.7)	ACF: 27 (29.3); External jugular vein: 2 (2.2); Forearm: 55 (59.8); Hand: 5 (5.4); Upper arm: 3 (3.3)
Cardenas-Garcia et al, [7] 2015	398 (54)	NR	Patients admitted to the MICU requiring vasoactive medication	NR	SAPS II: 75 (±15)	Hemodynamic instability/shock	Norepinephrine: 506 (65); Dopamine: 101 (13); Phenylephrine: 176 (22)	Norepinephrine: 0.70 mcg/kg/min (±0.2); Dopamine: 12.7 mcg/kg/min (±5.2); Phenylephrine: 3.25 mcg/kg/min (±1.7)	18G: 192 (25); 20G: 590 (75); 22G: 1 (0.1, deviation from protocol)	Upper extremity (majority in upper arm basilic or cephalic vein, though not systematically recorded)
Christensen et al, [8] 2024	556 (55.4)	26.8 (±5.1)	Adult patients scheduled for	No consent found or already have CVC	NR	Anesthesia-induced hypotension	Norepinephrine: 1004 (100)	8 or 40 mcg /ml	14G: 1 (0.1); 16G: 4 (0.4);	Arm fold: 123 (12.3);

© 2026 ZhangJian SJ et al. *JAMA Network Open*.

Author, year	Male (%)	BMI[a]	Inclusion criteria	Exclusion criteria	Illness severity[a]	Reason for vasopressor	Vasopressor medication, No. (%)	Vasopressor dose[a]	Gauge, No. (%)	Location, No. (%)
			general anesthesia and/or a central neural block						17G: 56 (5.6); 18G: 385 (38.3); 20G: 435 (43.3); 22G: 114 (11.4); Missing: 9 (0.9)	Forearm: 109 (10.9); Hand: 715 (71.3); Lower leg: 4 (0.4); Missing: 46 (4.6); Overarm: 7 (0.7)
Dansereau et al, [9] 2024	153 (61.2)	29.3 (±8.0)	Adult patients who were expected to require vasopressor therapy for less than 72hr, had two PIV lines, and were receiving low to moderate doses of vasopressors.	Cannot obtain 2 PIV lines, already have CVC, PIV sites did not have brisk blood return, using metacarpal area for vasopressor	Mortality rate: 44.4%	Various types of shock	Norepinephrine: 226 (59.6); Phenylephrine: 139 (36.7); Epinephrine: 9 (2.4); Vasopressin: 5 (1.3)	NR	NR	Basilic vein: 38 (10); Cephalic vein: 213 (56); Median cubital vein: 82 (21); Median vein: 46 (12); Others (1 femoral vein, 1 jugular vein, 1 midline catheter): 3 (1)
Datar et al, [10] 2018	129 (47)	27 (23-31)	Adults admitted to the Neuro ICU receiving phenylephrine infusion via PIV	Patients who received phenylephrine only through CVCs	NR	Hemodynamic augmentation: 110 (40); Postoperative hypotension: 89 (32); Hypotension from other causes: 61 (22); Sepsis: 17 (6)	Phenylephrine: 277 (100)	Maximum rate: 1.04 mcg/kg/min (±0.74)	16G: 13 (5); 18G: 98 (35); 20G: 113 (41); 22G: 5 (2); Unknown: 48 (17)	Others: 3 (1); Unknown: 48 (17); Upper extremity: 139 (50); Wrist/hand: 87 (32)

© 2026 ZhangJian SJ et al. *JAMA Network Open*.

Author, year	Male (%)	BMI[a]	Inclusion criteria	Exclusion criteria	Illness severity[a]	Reason for vasopressor	Vasopressor medication, No. (%)	Vasopressor dose[a]	Gauge, No. (%)	Location, No. (%)
Delaney et al, [11] 2019	233 (59.9)	NR	ARISE trial participants who received vasopressors for at least 30 minutes from ED presentation to 6 hours post-randomization	Patients without primary outcome data	APACHE-II: 17 (13-23)	Early septic shock	Norepinephrine: 293 (75); Metaraminol: 58 (15); Epinephrine: 38 (10)	NR	NR	NR
Delgado et al, [12] 2016	11 (55)	NR	Necessity for augmentation of cerebral or spinal perfusion pressure as determined by the neurointensivist.	NR	NR	Hemodynamic support or augmentation	Phenylephrine: 20 (100)	Peak dose: 0.7 mcg/kg/min (0.3-2) Average dose: 0.5 mcg/kg/min (0.2-1.8)	18G or larger: 19 (95); 20G: 1 (5)	Upper extremity (proximal to wrist): 20 (100)
Fabick et al, [13] 2023	1265 (58.5)	NR	Adult patients admitted to the ICU and received continuous vasopressor infusion for≧1 hour	Patients receiving vasopressors for code response or push-dose indications; Vasopressors used for organ procurement; No IV line information;	NR	Various types of shock	Norepinephrine; Epinephrine; Dopamine; Phenylephrine; Vasopressin	Median rate: Norepinephrine: 11.9 mcg/min (±15.5); Phenylephrine: 76.0 mcg/min (±47.3); Vasopressin: 0.04 U/min; Epinephrine: 0.1 mcg/kg/min	14G: 9 (0.4); 16G: 98 (4.5); 18G: 1140 (52.7); 20G: 833 (38.5); 22G: 76 (3.5); 24G: 1 (0.04); Unknown: 6 (0.3)	ACF: 718 (33.3); Basilic vein: 29 (1.3); Brachial vein: 44 (2.0); Cephalic vein: 25 (1.2); External jugular vein: 29 (1.3);

© 2026 ZhangJian SJ et al. *JAMA Network Open*.

Author, year	Male (%)	BMI[a]	Inclusion criteria	Exclusion criteria	Illness severity[a]	Reason for vasopressor	Vasopressor medication, No. (%)	Vasopressor dose[a]	Gauge, No. (%)	Location, No. (%)
				CVC placement before hospital presentation				(±0.06); Dopamine: 6.6 mcg/kg/min (±3.5)		Foot: 4 (0.2); Forearm: 725 (33.7); Hand: 212 (9.8); Intraosseous: 1 (0.1); Upper arm: 293 (13.6); Wrist: 74 (3.4)
Feng et al, [14] 2021	39 (33.6)	NR	Adults admitted to ICU requiring PIV norepinephrine	Receiving PIV norepinephrine < 1 hr	APACHE-II: 17.8 (±5.2)	Various types of shock	Norepinephrine: 116 (100)	NR	18G: 11 (7.5); 20G: 132 (89.8); 22G: 4 (2.7)	Antecubital vein: 116 (78.9); Arm vein: 18 (12.2); Median cubital vein: 13 (8.8)
Gandotra et al, [15] 2023	1111 (60.1)	NR	Adult patients with septic shock	Patients receiving vasopressors before ICU admission	SOFA: 10.4 (±3.4)	Septic shock	Norepinephrine (equivalents)	Maximum rate: 0.03 mcg/kg/min	NR	NR
Gershengorn et al, [16] 2023	142 (49.5)	28.2 (23.9-36.0)	Adult patients admitted to ICU	Under 18 yr; pregnant; admitted to non-medicine service or under observation status	NR	Various types of shock	Norepinephrine; Dopamine; Epinephrine; Vasopressin; Phenylephrine; Dobutamine	NR	NR	Basilic: 116 (40.4); Brachial: 118 (41.1); Others: 53 (18.4)
Groetzinger et al, [17] 2022	42 (48.3)	NR	Patients receiving peripheral norepinephrine	Patients with CVC already in place	SOFA: 8 (6-11)	Septic: 54 (62); Hemorrhagic: 9 (10); Medication	Norepinephrine: 87 (100)	Maximum rate: 0.1 mcg/kg/min (0.1-0.2)	16G: 7 (8); 18G: 46 (53); 20G: 25 (29);	ACF: 18 (21) Bicep: 35 (40) Forearm: 3 (3)

© 2026 ZhangJian SJ et al. *JAMA Network Open*.

Author, year	Male (%)	BMI[a]	Inclusion criteria	Exclusion criteria	Illness severity[a]	Reason for vasopressor	Vasopressor medication, No. (%)	Vasopressor dose[a]	Gauge, No. (%)	Location, No. (%)
						induced/OD: 5 (6); Obstructive/cardiogenic: 7 (8); Peri-procedural: 3 (3); Other/mixed 9 (10)			Other: 9 (10)	Not reported: 7 (8) Other: 7 (8) Upper extremity: 17 (20)
Hallengren et al, [18] 2017	50 (63)	NR	Age≧18yr; diagnosis of septic shock; norepinephrine administration, treatment in the IMCU	Patients treated in the ICU during the same hospitalization	APACHE-II: 26 (23-32)	Septic shock	Norepinephrine: 79 (100)	Maximum rate 0.2 mcg/kg/min	NR	NR
Han et al, [19] 2024	188 (68.9)	22.9 (20.4-25.3)	Patients with cerebrospinal disease who received metaraminol	Age<18yr; pregnancy; contraindication to metaraminol; bolus injection; CVC used; combined with other vasopressors; incomplete data	APACHE-II: 21 (16-27)	Mostly to enhance cerebral perfusion or prevent hypotension	Metaraminol: 273 (100)	Maximum rate: 8.7 ml/hr (5-10)	≧18G, commonly 20G	At external jugular or upper extremity veins, most common at right antecubital area
He et al, [20] 2022	380 (59.4)	NR	Adult patients with norepinephrine infusion>1hr	Contraindicated to norepinephrine; norepinephrine infusion <1hr	NR	Septic shock	Norepinephrine: 640 (100)	NR	<20G: 479 (74.8); ≧20G: 161 (25.2)	Forearm: 376 (58.8); Median cubital fossa: 80 (12.5); Upper arm: 184

© 2026 ZhangJian SJ et al. *JAMA Network Open*.

Author, year	Male (%)	BMI[a]	Inclusion criteria	Exclusion criteria	Illness severity[a]	Reason for vasopressor	Vasopressor medication, No. (%)	Vasopressor dose[a]	Gauge, No. (%)	Location, No. (%)
										(28.8);
Johnson et al, [21] 1977	NR	NR	Patient with gastric or esophageal varices	NR	30-day mortality: 45%	Hemorrhagic shock	Vasopressin: 11 (100)	0.1-0.4 U/min	NR	NR
Karlsson et al, [22] 2024	281 (60)	26 (23-31)	Patients with septic shock who received norepinephrine through a midline catheter	Patients who had a concurrent central line with no documentation of norepinephrine administration via midline catheter	NEWS 2: 8 (6-10) and In-hospital mortality: 32%	Septic shock	Norepinephrine: 472 (100)	Median dose: 0.12 mcg/kg/min (0.08-0.20)	18G: 463 (98); 20G: 9 (2)	Cephalic vein: 43 (10)<br>Basilic vein: 305 (69)<br>Brachial vein: 95 (21)
Kilian et al, [23] 2022	19 (55.9)	NR	Adult patients with septic shock admitted via ED and received vasopressors	Mixed or non-septic shock; hospital transfers; history of heart failure	NR	Septic shock	Norepinephrine: 29 (85.3); Phenylephrine: 3 (8.8); Epinephrine: 2 (5.9); Vasopressin: 0 (0)	Maximum rate: Norepinephrine: 0.2 mcg/kg/min	18 or 20G	Proximal to ACF: 21 (61.9)<br>Distal to ACF: 10 (29.3)<br>Unknown: 3 (8.8)
Lewis et al, [24] 2019	107 (53)	25 (21-29)	Adult patients receiving vasopressors through a PIV in the ICU	CVC in place at vasopressor initiation and vasopressor infusion <1hr	APACHE-II: 21 (17-25)	Septic shock: 147 (73); Cardiogenic shock: 28 (14); Stroke/neurological disorder: 15 (7); Others: 12 (6)	Norepinephrine: 146 (72)[b]; Phenylephrine: 73 (36)[b]; Vasopressin: 4 (2)[b]; Epinephrine: 2 (1)[b]; Dopamine: 2 (1)[b]	Maximum rate Norepinephrine: 0.13 mcg/kg/min (0.08-0.3); Phenylephrine: 95 mcg/min (50-150); Vasopressin: 0.04	18G: 46 (23)[b]; 20G: 149 (74)[b]; 22G: 103 (51)[b]; Others: 6 (3)[b]	ACF: 109 (54)[b]<br>Forearm: 145 (72)[b]<br>Hand: 81 (40)[b]<br>Others: 5 (2)[b]

© 2026 ZhangJian SJ et al. *JAMA Network Open*.

Author, year	Male (%)	BMI[a]	Inclusion criteria	Exclusion criteria	Illness severity[a]	Reason for vasopressor	Vasopressor medication, No. (%)	Vasopressor dose[a]	Gauge, No. (%)	Location, No. (%)
								U/min (0.04-0.06); Epinephrine: 0.06mcg/kg/min; Dopamine: 9 mcg/kg/min		
Marques et al, [25] 2022	29 (45)	NR	Adult patients receiving peripheral vasopressors for >1hr	Patients receiving peripheral vasopressors for <1hr; pregnant women or without consent	NR	Various types of shock	Epinephrine: 42 (66)[b]; Norepinephrine: 26 (41)[b]; Dopamine: 7 (11)[b]; Dobutamine: 1 (2)[b]	NR	16 or 18G: 37 (58); 20 or 22G: 26 (41); Unknown: 1 (2)	Antecubital/upper arm: 20 (31); External jugular vein: 6 (9); Forearm: 10 (16); Hand/wrist: 17 (27); Lower extremities: 1 (2); Unknown: 10 (16)
Marti et al, [26] 2022	NR	NR	Adult patients requiring vasopressors peripherally and expected to require low to moderate doses of a single vasopressor for up to 72hr	Need for two or more vasopressors; Peripheral IV site not having brisk blood return; Limb restrictions. Inability to support two peripheral IV sites	NR	Blood pressure support	Norepinephrine: 67 (51.9); Phenylephrine: 58 (45); Vasopressin: 1 (0.8); Epinephrine: 3 (2.3)	Maximum rate: Norepinephrine: 14.9 mcg/min; Phenylephrine: 119.9 mcg/min; Vasopressin: 0.03 U/min; Epinephrine: 0.3 mcg/kg/min	NR	NR
McCurry et al, [27]	49 (61.3)	26.7 (23.3-31.5)	Adult≧18yr	Patient receive	In -hospital	Mixed shock	Vasopressin: 80	0.01-0.08 U/min	16G: 2 (2.5);	Above ACF: 11

© 2026 ZhangJian SJ et al. *JAMA Network Open*.

Author, year	Male (%)	BMI[a]	Inclusion criteria	Exclusion criteria	Illness severity[a]	Reason for vasopressor	Vasopressor medication, No. (%)	Vasopressor dose[a]	Gauge, No. (%)	Location, No. (%)
2025			admitted to ED and received peripheral vasopressin infusion	vasopressin from midline or CVC or if peripheral vasopressin administration cannot be verified	mortality: 53.8%		(100)		18G: 41 (51.3); 20G: 12 (15); 22G: 5 (6.3); Unknown: 20 (25)	(13.8); ACF: 34 (42.5); Forearm: 18 (22.5); Non-arm: 5 (6.3); Unknown: 6 (7.5); Wrist: 6 (7.5)
Medlej et al, [28] 2018	34 (61.8)	NR	Patients presenting to the ED with circulatory shock requiring vasopressor infusion through a PIVC	NR	In hospital mortality: 32%	Circulatory shock	Norepinephrine: 50 (91); Dopamine: 5 (9)	Maximum rate Norepinephrine: 30 mcg/min, Dopamine: 15 mcg/kg/min	16G: 6 (10.9); 18G: 20 (36.4); 20G: 28 (50.9); 22G: 1 (1.8)	ACF: 22 (40); External jugular: 2 (3.6); Forearm: 10 (18.2); Hand: 20 (36.4); Upper arm: 1 (1.8)
Messina et al, [29] 2021	64 (50.4)	24 (21-26)	Patients with septic shock receiving norepinephrine peripherally in the ED	Patients who died within 1 hour of ED admission and patients with missing data on norepinephrine dose and timing	SOFA: 4 (2-5); Charlson Comorbidity Index: 2 (1-3)	Septic shock	Norepinephrine: 127 (100)	Maximum rate: 0.1 mcg/kg/min (0.07-0.16)	NR	NR
Munroe et al, [30] 2024	208 (52)	27.7 (22.5-33.2)	Adult patients with vasopressor initiation within 6 hours of hospital arrival	Pregnancy; patients younger than 18 yr; transferred patients	Predicted mortality score: 0.4 (0.3-0.6)	Hypotension (SBP <90 or MAP<65, or SBP< 100 with baseline HTN)	Norepinephrine: 337 (84.3); Phenylephrine: 22 (5.5); Epinephrine:	NR	NR	NR

© 2026 ZhangJian SJ et al. *JAMA Network Open*.

Author, year	Male (%)	BMI[a]	Inclusion criteria	Exclusion criteria	Illness severity[a]	Reason for vasopressor	Vasopressor medication, No. (%)	Vasopressor dose[a]	Gauge, No. (%)	Location, No. (%)
							28 (7); Dopamine: 13 (3.3)			
Munroe et al, [31] 2025	315 (54.1)	NR	Received vasopressors within 24hr of trial enrollment	Route of vasopressor administration was unknown or they had central access present prior to enrollment	Charlson comorbidity index: 4 (2-6)	Septic shock	NR	NR	NR	NR
Nguyen et al, [32] 2021	104 (58.8)	NR	Adult patients receiving peripheral norepinephrine in the ED	Pregnant individuals; prisoners; patients in cardiac arrest; patients receiving pre-hospital vasopressors or multiple vasopressors in the ED; cases initiated outside the ED or in unclear circumstances	APACHE-II: 25 (19-34)	Distributive shock: 111 (62.7); Peri-procedural/peri-intubation: 20 (11.3); Hypovolemic shock: 19 (10.7); Neurogenic shock: 11 (6.2); Cardiogenic shock: 8 (4.5); Obstructive shock: 1 (0.6)	Norepinephrine: 177 (100)	Maximum rate: 10 mcg/min (5-16.5)	14G: 2 (1.1); 16G: 15 (8.5); 18G: 103 (58.2); 20G: 45 (25.4); 22G: 1 (0.6); Unknown: 11 (6.2)	ACF: 121 (68.4); Arm (distal to ACF): 33 (18.6); External jugular vein: 16 (9); Hand: 7 (4)
Padmanaban et al, [33] 2020	66 (54)	24.1 (±4.4)	Patients receiving vasopressors through a PIVC for more	Patients with a CVC at the time of vasopressor	Septic shock: 89 (73); Cardiogenic shock: 7 (6);	Hemodynamic emergencies	Norepinephrine: 118 (97)[b]; Vasopressin: 29 (24)[b];	Maximum rate Norepinephrine: 14.8 mcg/min	16G: 5 (4); 18G: 70 (57); 20G: 41 (34);	Cubital: 11 (9); External jugular vein: 44 (36);

© 2026 ZhangJian SJ et al. *JAMA Network Open*.

Author, year	Male (%)	BMI[a]	Inclusion criteria	Exclusion criteria	Illness severity[a]	Reason for vasopressor	Vasopressor medication, No. (%)	Vasopressor dose[a]	Gauge, No. (%)	Location, No. (%)
			than 1hr	initiation; patients receiving vasopressors for less than one hour; patients without secured PIVC access	Stroke/Subarachnoid hemorrhage: 12 (10); Others: 14 (11)		Epinephrine: 6 (5)[b]; Dopamine: 2 (2)[b]	(±8.8); Vasopressin: 2 U/hr (±0.3); Epinephrine: 10.5 mcg/min (±7.8); Dopamine: 10 mcg/kg/min	22G: 6 (5)	Forearm: 39 (32); Hand: 28 (23)
Pancaro et al, [34] 2020	NR	NR	Surgical patients receiving norepinephrine peripheral intravenous infusions	NR	NR	Perioperative hypotension	Norepinephrine: 14385 (100)	NR	NR	NR
Petros et al, [35] 2025	127 (50.8)	NR	Adult patients receiving PIV vasopressors for >1hr	Non-traumatic limb amputation or cardiac arrest prior to vasoactive initiation	Mortality: 57.6%	Mixed shock	Norepinephrine: 144 (57.6)[b]; Epinephrine: 104 (41.6)[b]; Dopamine: 17 (6.8)[b]	Median dose: Norepinephrine: 0.25 mcg/kg/min (0.2-0.5); Dopamine: 10 mcg/kg/min (7.5-27.5); Epinephrine: 0.3 mcg/kg/min (0.2-0.5)	18G: 147 (58.8); 20G: 59 (23.6); 22G: 43 (17.2); 24G: 1 (0.4)	ACF: 70 (28); External jugular: 12 (4.8); Foot: 2 (0.8); Forearm: 106 (42.4); Hand: 47 (18.8); Lower leg: 1 (0.4); Upper arm: 6 (2.4); Wrist: 6 (2.4)
Powell et al, [36] 2023	63 (64)	NR	Adult patients requiring	Initiation of norepinephrine prior	NR	Various types of shock	Norepinephrine: 98 (100)	Maximum rate: 0.08 mcg/kg/min	18G: 22 (22); 20G: 13 (13);	ACF: 54 (55); Upper arm (basilic

© 2026 ZhangJian SJ et al. *JAMA Network Open*.

Author, year	Male (%)	BMI[a]	Inclusion criteria	Exclusion criteria	Illness severity[a]	Reason for vasopressor	Vasopressor medication, No. (%)	Vasopressor dose[a]	Gauge, No. (%)	Location, No. (%)
			norepinephrine	to transfer; requirement for multiple vasopressors; hand/wrist IV access only; incomplete data				(0.05-0.15)	Unspecified: 63 (64)	vein): 4 (4); Upper forearm: 40 (41)
Prasanna et al, [37] 2021	117 (47.2)	28 (23-35)	Adult patients receiving vasopressors	NR	APACHE-II: 29 (23-34)	Septic/distributive shock: 122 (49.1); Neurogenic shock: 88 (35.6); Cardiogenic shock: 38 (15.3)	Norepinephrine: 165 (66.5)[b]; Phenylephrine: 158 (63.7)[b]; Vasopressin: 123 (49.6)[b]; Epinephrine: 56 (22.6)[b]; Angiotensin II: 6 (2.4)[b]	Maximum rate Norepinephrine: 47 mcg/min (0.67 mcg/kg/min); Phenylephrine: 350 mcg/min (5 mcg/kg/min); Epinephrine: 35 mcg/min (0.5 mcg/kg/min); Vasopressin: 0.08 U/min; Angiotensin II: 80 ng/kg/min	midline catheter: 4Fr 20cm	Axillary: 0 (0); Basilic: 245 (98.8); Brachial: 2 (0.8); Cephalic: 1 (0.4)
Putland et al, [38] 2006	88 (40)	NR	Adult patients admitted to ED with diagnosis of asthma receiving IV	Patients >55yr	Australian triage scale: category 1: 46 (21); category 2: 129 (59); category 3: 45	Severe asthma	Epinephrine: 220 (100)	1.5 mcg/min (0.5-13.3)	NR	NR

© 2026 ZhangJian SJ et al. *JAMA Network Open*.

Author, year	Male (%)	BMI[a]	Inclusion criteria	Exclusion criteria	Illness severity[a]	Reason for vasopressor	Vasopressor medication, No. (%)	Vasopressor dose[a]	Gauge, No. (%)	Location, No. (%)
			epinephrine infusion		(20)					
Ramanan et al, [39] 2025	20 (50)	NR	Adult patients requiring vasopressors	<18yr; pregnancy; prior vasopressor use; high-dose norepinephrine (>0.1mcg/kg/min); multiple vasopressors; existing CVC; or CVC required for specific therapy	30 days mortality: early: 15%; late: 5%	NR	Norepinephrine: 40 (100)[b]; Epinephrine: 6 (16)[b]; Vasopressin: 6 (16)[b]	Maximum rate: Norepinephrine: 0.10 mcg/kg/min (0.08-0.17); Epinephrine: 0.15 mcg/kg/min (0.05-0.27); Vasopressin: 0.04 U/min	NR	NR
Ruchti et al, [40] 2021	47 (47)	NR	Patients without central venous line requiring low-dose vasopressor support	NR	APACHE-II: Noradrenaline group: 14 (9-16); Metaraminol group: 11 (8-17)	Various types of shock	Norepinephrine: 50 (50); Metaraminol: 50 (50)	Maximum rate Norepinephrine: 600 mcg/hr; Metaraminol: >5 mg/hr	Ideally 18G or larger	NR
Sardaneh et al, [41] 2021	38 (47)	NR	Patients admitted to the ICU during the study period who received metaraminol intravenous infusions for management of	Vasopressors used for indications other than shock. Transfer from another hospital. Metaraminol not used for the initial episode of shock	APACHE-II: 62 (49-76)	Various types of shock	Metaraminol: 81 (100)	Maximum rate: 3.0 mg/hr (2.5-5.0)	NR	NR

© 2026 ZhangJian SJ et al. *JAMA Network Open*.

Author, year	Male (%)	BMI[a]	Inclusion criteria	Exclusion criteria	Illness severity[a]	Reason for vasopressor	Vasopressor medication, No. (%)	Vasopressor dose[a]	Gauge, No. (%)	Location, No. (%)
			shock							
Schmucki et al, [42] 2025	NR	26 (12-61)	Adult patients received surgery	Without consent or missing data	Mostly ASA 2 or 3	Intra-operative hypotension	Norepinephrine: 1561 (100)	Norepinephrine: 4 mcg/min (0-24.3)	NR	NR
Shyu et al, [43] 2025	2061 (55.2)	28.4 (24.4-33.5)	Adult patients receiving PIV vasopressors	Inotrope-only therapy, vasopressors given only in OR/ED or as bolus, dopamine use, vasopressor duration <1hr, inter-hospital transfers, and prescheduled surgical admissions	Hospital mortality: 31%	NR	Norepinephrine: 3303 (88); Phenylephrine: 270 (7.2); Epinephrine: 137 (3.7); Vasopressin: 24 (0.6)	NR	NR	NR
Spiegel et al, [44] 2020	NR	NR	NR	NR	NR	Various types of shock	NR	NR	5 Fr catheter specifically use for vasopressor	Upper extremities
Stolz et al, [45] 2022	104 (53.6)	29.3 (±6.8)	Adult patients admitted to ICU requiring vasopressor	Patients who received bolus doses of vasopressors only	APACHE-III: 70.1 (±26.6)	Various types of shock	Norepinephrine; Epinephrine; Metaraminol; Phenylephrine	Norepinephrine: 0.99 mcg/kg/min (0.6-1.64); Epinephrine: 0.09 mcg/kg/min (0.08-0.4); Metaraminol: 0.31 mcg/kg/min (0.25-	NR	NR

© 2026 ZhangJian SJ et al. *JAMA Network Open*.

Author, year	Male (%)	BMI[a]	Inclusion criteria	Exclusion criteria	Illness severity[a]	Reason for vasopressor	Vasopressor medication, No. (%)	Vasopressor dose[a]	Gauge, No. (%)	Location, No. (%)
								0.5); Phenylephrine: 0.17 mcg/kg/min (0.09-0.27)		
Vitharana et al, [46] 2023	29 (55.8)	NR	Adult patients admitted with sepsis and requiring vasopressors	Pregnant patients and children <15yr	NR	Sepsis-induced hypotension	Norepinephrine: 52 (100)	Varied from 0.05 to 0.3 mcg/kg/min	16G: 3 (6.3); 18G: 28 (58.3); 20G: 14 (29.2); 22G: 3 (6.3)	ACF: 2 (4.2); Arm: 2 (4.2); Dorsum of the hand: 18 (37.5); External jugular: 2 (4.2); Forearm: 24 (50)
Yasuda et al, [47] 2022	NR	NR	Adult patients admitted to the ICU with newly inserted PIVCs after ICU admission	PIVCs inserted before ICU admission	NR	NR	Norepinephrine; Dobutamine	NR	NR	NR
Yerke et al, [48] 2024	NR	28.2 (23.7-33.6)	Adult patients received norepinephrine via PIVC	Patients requiring multiple vasopressors	NR	Hypotension or shock	Norepinephrine: 635 (100)	Norepinephrine: 10 mcg/min (6-15)	18G; 20G; 22G	Above wrist, below ACF
Zichichi et al, [49] 2024	111 (56)	26.9 (23-32.3)	Adult patients received vasopressor ≧1hr	Preexisting CVC access	NR	Various types of shock	Norepinephrine: 142 (71.7); Phenylephrine: 48 (24.2); Epinephrine:	Norepinephrine: 0.13 mcg/kg/min (0.06-0.3), Phenylephrine: 1.1	16G: 5 (2.7); 18G: 71 (35.8); 20G: 93 (47); Other: 23 (11.6);	ACF: 62 (50.3); Forearm: 24 (30); Metacarpal: 42 (39.6);

© 2026 ZhangJian SJ et al. *JAMA Network Open*.

Author, year	Male (%)	BMI[a]	Inclusion criteria	Exclusion criteria	Illness severity[a]	Reason for vasopressor	Vasopressor medication, No. (%)	Vasopressor dose[a]	Gauge, No. (%)	Location, No. (%)
							8 (4.0)	mcg/kg/min (0.7-2.8), Epinephrine: 0.21 mcg/kg/min (0.08-1)	No document: 6 (3)	Other: 34 (26.7); Upper extremity: 36 (53.5)

Abbreviations: ACF: antecubital fossa; APACHE II: Acute Physiology and Chronic Health Evaluation II; ASA: American Society of Anesthesiologists; BP: blood pressure; BMI: body mass index; NA: not applicable; CVC: central venous catheter; ED: emergency department; HTN: hypertension; ICU: intensive care unit; IMCU: intermediate care unit; IV: intravenous; MAP: mean arterial pressure; MAOI: Monoamine oxidase inhibitor; MEWS: Modified Early Warning Score; MICU: medical intensive care unit; NEWS: national early warning score; OD: over-dosed; OR: operation room; PIV: peripheral intravenous; PIVC: peripheral intravenous catheter; qSOFA: quick Sequential Organ Failure Assessment; SAPS II: Simplified Acute Physiology Score II; SBP: systolic blood pressure; SOFA: Sequential Organ Failure Assessment

[a] Data presented as “mean (± standard deviation)” or “median (interquartile)” or “median (range)”

[b] Numbers represent the number of catheters rather than the number of patients; therefore, the percentages exceed 10

© 2026 ZhangJian SJ et al. *JAMA Network Open*.

eTable 3. Quality Assessment of Prevalence Studies Based on the JBI Checklist

Study	Q1	Q2	Q3	Q4	Q5	Q6	Q7	Q8	Q9	Total	Overall ROB
Asher et al, 2023	Y	Y	Y	Y	Y	U	U	N	Y	6	U
Ballieu et al, 2021	Y	N	Y	Y	Y	U	Y	N	Y	6	U
Bima et al, 2022	Y	Y	N	Y	Y	U	U	N	Y	5	H
Cape et al, 2022	Y	Y	Y	Y	Y	Y	Y	N	Y	8	L
Cardenas-Garcia et al, 2015	Y	Y	Y	Y	Y	U	Y	N	Y	7	L
Christensen et al, 2024	Y	Y	Y	Y	Y	U	U	Y	Y	7	L
Dansereau et al, 2024	Y	Y	Y	Y	Y	U	U	N	Y	6	U
Datar et al, 2018	Y	Y	Y	Y	Y	U	U	N	Y	6	U
Delaney et al, 2020	Y	Y	Y	Y	Y	U	U	N	Y	6	U
Delgado et al, 2016	Y	Y	N	Y	Y	U	Y	N	Y	6	U
Fabick et al, 2023	Y	Y	Y	Y	Y	Y	Y	N	Y	8	L
Feng et al, 2021	Y	Y	Y	Y	Y	U	U	N	Y	6	U
Gandotra et al, 2023	Y	Y	Y	N	U	Y	Y	Y	Y	7	U
Groetzinger et al, 2022	Y	Y	Y	Y	Y	Y	Y	N	Y	8	L
Hallengren et al, 2017	Y	Y	Y	Y	Y	U	U	N	Y	6	U
Han et al, 2024	Y	Y	Y	Y	Y	U	U	N	Y	6	U
He et al, 2022	Y	Y	Y	Y	Y	U	U	N	Y	6	U
Kilian et al, 2022	Y	Y	N	Y	Y	U	U	N	Y	5	U
Lewis et al, 2019	Y	Y	Y	Y	Y	Y	U	N	Y	7	L
Marques et al, 2022	Y	N	Y	Y	Y	Y	Y	Y	Y	8	L

© 2026 ZhangJian SJ et al. *JAMA Network Open*.

Marti et al, 2022	Y	Y	Y	N	U	U	Y	N	Y	5	L
Medlej et al, 2018	Y	Y	Y	Y	U	U	U	N	Y	5	U
Messina et al, 2021	Y	Y	Y	Y	Y	U	U	N	Y	6	U
Munroe et al, 2024	Y	Y	Y	Y	Y	U	U	N	Y	6	U
Nguyen et al, 2021	Y	Y	Y	Y	Y	U	Y	N	Y	7	L
Padmanaban et al, 2020	Y	Y	Y	Y	Y	Y	Y	N	Y	8	L
Pancaro et al, 2020	Y	Y	Y	Y	Y	Y	U	Y	Y	8	L
Petros et al, 2025	Y	Y	Y	Y	Y	Y	Y	N	Y	8	L
Powell et al, 2023	Y	Y	Y	Y	Y	U	Y	N	Y	7	L
Putland et al, 2006	Y	Y	Y	Y	U	U	U	Y	Y	6	U
Ruchti et al, 2021	Y	Y	Y	Y	Y	U	U	N	Y	6	U
Sardaneh et al, 2021	Y	Y	Y	Y	Y	U	U	N	Y	6	U
Schmucki et al, 2025	Y	Y	Y	N	U	U	U	N	Y	4	H
Shyu et al, 2025	Y	Y	Y	Y	Y	U	U	N	Y	6	U
Stolz et al, 2022	Y	N	Y	Y	Y	U	U	N	Y	5	H
Vitharana et al, 2023	U	Y	Y	N	U	U	U	N	Y	3	H
Yasuda et al, 2022	Y	Y	Y	Y	Y	Y	U	N	Y	7	L
Yerke et al, 2024	Y	Y	Y	Y	Y	Y	Y	Y	Y	9	L
Zichichi et al, 2024	Y	Y	Y	Y	Y	U	Y	N	Y	7	L

Abbreviations: Y, yes; N, no; U, unclear; L, low; H, high; ROB, risk of bias

Questions from JBI Critical Appraisal Checklist for Studies Reporting Prevalence Data

1. Was the sample frame appropriate to address the target population?

2. Were study participants sampled in an appropriate way?

3. Was the sample size adequate?

4. Were the study subjects and the setting described in detail?

© 2026 ZhangJian SJ et al. *JAMA Network Open*.

5. Was the data analysis conducted with sufficient coverage of the identified sample?

6. Were valid methods used for the identification of the condition?

7. Was the condition measured in a standard, reliable way for all participants?

8. Was there appropriate statistical analysis?

9. Was the response rate adequate, and if not, was the low response rate managed appropriately?

© 2026 ZhangJian SJ et al. *JAMA Network Open*.

eTable 4. Assessment of Risk of Bias in Randomized Trials Using the Revised Cochrane RoB 2 Tool

STUDY	Domain 1	Domain 2	Domain 3	Domain 4	Domain 5	Total
Aykanat et al, 2022	Low	Low	Low	Low	Low	Low
Andrews et al, 2017	Low	Low	Low	Low	Low	Low
Ramanan et al, 2025	Low	High	Low	High	Low	High
Johnson et al, 1977	Some	High	Low	Low	Low	High

Low= Low risk of bias in that domain, Some= some concerns in that domain, High= High risk of bias in that domain

Domains of Revised Cochrane risk-of-bias tool for randomized trials:

Domain 1: Risk of bias arising from the randomization process

Domain 2: Risk of bias due to deviations from the intended interventions

Domain 3: Risk of bias due to missing outcome data

Domain 4: Risk of bias in measurement of the outcome

Domain 5: Risk of bias in selection of the reported result

© 2026 ZhangJian SJ et al. *JAMA Network Open*.

eTable 5. Details of adverse event features

Author, year	Total number of AE	Age (years)[a]	Male (%)	Vasopressor dose[a]	Vasopressor duration[a]	Gauge, No. (%)	Location, No. (%)	AE detail per vasopressor medication						Intervention for AE
								**Norepinephrine**	**Dopamine**	**Epinephrine**	**Phenylephrine**	**Vasopressin**	**Metaraminol**	
Andrews et al, [1] 2017	0	NA	NA	NA	NA	NA	NA	NA	0% (0/17)	NA	NA	NA	NA	NA
Asher et al, [2] 2023	7	NR	NR	NR	NR	20G: 7 (100)	Above wrist: 7 (100)	NR	NR	NR	NR	NR	NA	All AEs were treated conservatively
Aykanat et al, [3] 2022	3	NR	NR	NR	NR	NR	NR	Skin color change: 10% (3/30)	NA	NA	NA	NA	NA	NR
Ballieu et al, [4] 2021	9	NR	NR	NR	NR	18G or larger	NR	NA	NA	NA	Local skin erythema/swelling: 4.8% (6/125); Extravasation: 1.6% (2/125); Thrombophlebitis: 0.8% (1/125)	NA	NA	Removal of the PIVC; heat application and elevation
Bima et al, [5] 2022	0	NA	NA	NA	NA	NA	NA	NA	0	NA	NA	NA	NA	NA
Cape et al, [6] 2022	3	NR	NR	Norepinephrine 0.6; 0.3; 0.8 mcg/kg/min	2hr44min (2hr42min to 2hr45min)	20G: 3 (100)	Forearm: 3 (100)	Infiltration: 3.3% (3/92)	NA	NA	NA	NA	NA	All infiltration treated with phentolamine
Cardenas-	19	NR	NR	NR	NR	<20G: 1 (5);	NR	Extravasation:	Extravasatio	NA	0% (0/176)	NA	NA	Phentolamine injection

© 2026 ZhangJian SJ et al. *JAMA Network Open*.

Author, year	Total number of AE	Age (years)[a]	Male (%)	Vasopressor dose[a]	Vasopressor duration[a]	Gauge, No. (%)	Location, No. (%)	AE detail per vasopressor medication						Intervention for AE
								Norepinephrine	Dopamine	Epinephrine	Phenylephrine	Vasopressin	Metaraminol	
Garcia et al, [7] 2015						≧20G: 18 (95)		3.2% (16/506)	ns: 3.0% (3/101)					and application of nitroglycerin paste at extravasation site
Christensen et al, [8] 2024	110	71.0 (±12.0)	NR	NR	NR	NR	NR	Extravasation: 2.3% (23/1004); Paleness of skin: 8.7% (87/1004)	NA	NA	NA	NA	NA	Change to another PIV line; No phentolamine used
Dansereau et al, [9] 2024	14	72.1 (±15.6)	10 (71.4)	Norepinephrine: 11.1 mcg/min (±5.5); Phenylephrine: 126.7 mcg/min (±52.5)	Norepinephrine: 46.3h (±30.0); Phenylephrine: 39.3h (±41.3)	18G: 2 (14.3); 20G: 5 (35.7); 22G: 7 (50)	Basilic: 2 (14.3); Cephalic: 7 (50); Median: 3 (21.4); Median cubital: 2 (14.3)	Phlebitis: 2.2% (5/227); Infiltration: 2.6% (6/227)	NA	0	Phlebitis: 2.2% (3/139); Infiltration: 0.7% (1/139)	0	NA	No treatment needed: 5; Line removed: 7; Warm compression: 2; Phentolamine injection: 2; PIV site switch: 3
Datar et al, [10] 2018	9	NR	5 (56)	NR	NR	NR	NR	NA	NA	NA	Infiltration: 3.2% (9/277)	NA	NA	None
Delaney et al, [11] 2019	0	NA	NA	NA	NA	NA	NA	0% (0/293)	NA	0% (0/38)	NA	NA	0% (0/58)	NA
Delgado et al, [12] 2016	1	49	0 (0)	Phenylephrine Peak dose: 0.35 mcg/kg/min;	17.3h	18G: 1 (100)	ACF: 1 (100)	NA	NA	NA	Pain, erythema, swelling, and need to replace	NA	NA	Replace IV site

© 2026 ZhangJian SJ et al. *JAMA Network Open*.

Author, year	Total number of AE	Age (years)[a]	Male (%)	Vasopressor dose[a]	Vasopressor duration[a]	Gauge, No. (%)	Location, No. (%)	AE detail per vasopressor medication						Intervention for AE
								Norepinephrine	Dopamine	Epinephrine	Phenylephrine	Vasopressin	Metaraminol	
				Mean dose: 0.33 mcg/kg/min							IV: 5% (1/20)			
Fabick et al, [13] 2023	58	NR	NR	NR	NR	NR	NR	Extravasation: 2% (27/1340)	Extravasation: 4.1% (4/98)	0	Extravasation: 2.3% (19/815)	Extravasation: 0.5% (1/217)	NA	Phentolamine used: 14; Nitroglycerin used: 2
Feng et al, [14] 2021	6	NR	NR	Norepinephrine: 0.75 mcg/kg/hr (±0.04)	Norepinephrine: 29h (23~39)	20G: 5 (83.3); 22G: 1 (16.7)	ACF: 1 (16.7); Arm vein: 1 (16.7); Median cubital vein: 4 (66.6)	Extravasation: 5.17% (6/116)	NA	NA	NA	NA	NA	Conservative treatment
Gershengorn et al, [16] 2023	34	NR	NR	NR	NR	NR	NR	NR	NR	NR	NR	NR	NA	NR
Groetzinger et al, [17] 2022	3	72; 56; 62	0	Norepinephrine Max dose 0.62; 0.1; 0.1 mcg/kg/min, Average dose: 0.5; 0.04; 0.06 mcg/kg/min	7.8h; 5.8h; 7.2h	NR; 18G; 18G	NR; upper extremity; upper extremity	Infiltration: 1.1% (1/87); Phlebitis: 2.3% (2/87)	NA	NA	NA	NA	NA	NA
Hallengren et al, [18] 2017	0	NA	NA	NA	NA	NA	NA	0% (0/79)	NA	NA	NA	NA	NA	NA

© 2026 ZhangJian SJ et al. *JAMA Network Open*.

Author, year	Total number of AE	Age (years)[a]	Male (%)	Vasopressor dose[a]	Vasopressor duration[a]	Gauge, No. (%)	Location, No. (%)	AE detail per vasopressor medication						Intervention for AE
								Norepinephrine	Dopamine	Epinephrine	Phenylephrine	Vasopressin	Metaraminol	
Han et al, [19] 2024	5	74.4 (69.0-80.5)	3 (60)	Metaraminol concentration started from 1.0 mg/ml, max infusion rate 20 mg/hr	88.4h (54.5-131.6)	NR	Upper arm: 4 (80); Forearm and ACF: 1 (20)	NA	NA	NA	NA	NA	Phlebitis: 1.8% (5/273); Infiltration: 0.4% (1/273)	All treated with local tissue treatment including administration of mucopolysaccharide polysulfate cream
He et al, [20] 2022	27	54.4 (±6.7)	380 (59.4)	Norepinephrine: >0.3 mcg/kg/min: 17; ≦0.3 mcg/kg/min: 10	Norepinephrine: >24h: 20; ≦24h: 7	< 20G: 16 (59.3); ≧20G: 11 (40.7)	Forearm: 15 (55.6); Upper arm: 9 (33.3); Medial cubital fossa: 3 (11.1)	Extravasation: 100% (27/27)	NA	NA	NA	NA	NA	NR
Johnson et al, [21] 1977	1	NR	NR	NR	NR	NR	Forearm: 1 (100)	NA	NA	NA	NA	Phlebitis 9% (1/11)	NA	NR
Karlsson et al, [22] 2024	46	NR	NR	NR	NR	NR	Either the brachial, cephalic or basilic vein	Extravasation: 0.2% (1/472); Infiltration/leakage: 1.9% (9/472); Pain/irritant: 6.1% (29/472); Thrombosis:1.5%	NA	NA	NA	NA	NA	Close monitor for extravasation; Anticoagulant for thrombosis

© 2026 ZhangJian SJ et al. *JAMA Network Open*.

Author, year	Total number of AE	Age (years)[a]	Male (%)	Vasopressor dose[a]	Vasopressor duration[a]	Gauge, No. (%)	Location, No. (%)	AE detail per vasopressor medication						Intervention for AE
								Norepinephrine	Dopamine	Epinephrine	Phenylephrine	Vasopressin	Metaraminol	
								(7/472)						
Kilian et al, [23] 2022	0	NA	NA	NA	NA	NA	NA	0	NA	0	0	0	NA	NA
Lewis et al, [24] 2019	8	75 (69.5-89)	3 (37.5)	Norepinephrine equivalents median dose at time of extravasation: 0.11 mcg/kg/min	21h (12-30)	<20G: 2 (25%); ≧20G: 6 (75)	Hand: 2 (25); ACF: 2 (25); Others: 4 (50)	Extravasation: 2.7% (4/146)	0% (0/2)	0% (0/2)	Extravasation: 5.5% (4/73)	0% (0/4)	NA	Conservative treatment
Marques et al, [25] 2022	2	NR	NR	NR	48h; 93h	18G; 20G	Wrist; another not documented	NR	NR	NR	NA	NA	NA	Treated with limb elevation and warm compression
Marti et al, [26] 2022	3	NR	NR	Norepinephrine: 1 event at 6 mcg/min; 1 event at 14 mcg/min; Phenylephrine: 1 event at 160 mcg/min	Norepinephrine: 1 event at: 28h; 1 event at 62h; Phenylephrine: 1 event at 76h	NR	NR	Possible extravasation: 2.5% (2/79)	0	0	Possible extravasation: 1.6% (1/79)	0	NA	No specific treatment required
McCurry et al, [27] 2025	0	NA	NA	NA	NA	NA	NA	NA	NA	NA	NA	0	NA	NA
Medlej et al, [28] 2018	3	42; 57; 58	1 (33)	Norepinephrine Maximal dose: 7	Norepinephrine: 11h; 28h; 40h	20G: 3 (100)	Hand: 2 (67);	Drug extravasation 6%	0% (0/5)	NA	NA	NA	NA	None

© 2026 ZhangJian SJ et al. *JAMA Network Open*.

Author, year	Total number of AE	Age (years)[a]	Male (%)	Vasopressor dose[a]	Vasopressor duration[a]	Gauge, No. (%)	Location, No. (%)	AE detail per vasopressor medication						Intervention for AE
								Norepinephrine	Dopamine	Epinephrine	Phenylephrine	Vasopressin	Metaraminol	
				mcg/min: 2 (67); 19 mcg/min: 1 (33)			ACF: 1 (33)	(3/50) causing: Skin pallor (1/50); Non-blanching skin erythema (1/50); Thrombophlebitis (1/50)						
Messina et al, [29] 2021	5	NR	NR	NR	NR	NR	NR	Confirmed extravasation: 0.8% (1/127); Possible extravasations: 3.1% (4/127)	NA	NA	NA	NA	NA	NR
Munroe et al, [30] 2024	0	NA	NA	NA	NA	NA	NA	0	0	0	0	0	NA	NR
Munroe et al, [31] 2025	3	NR	NR	NR	NR	NR	NR	NR	NR	NR	NR	NR	NR	NR
Nguyen et al, [32] 2021	8	64.5 (47-79)	5 (62.5%)	Norepinephrine Max dose: 6.5 mcg/min (5-10)	Norepinephrine: 105min (37.5-170.5)	18G: 4 (50); 16G: 1 (12.5); 20G: 3 (37.5),	ACF: 4 (50); External jugular vein: 1 (12.5); Arm: 3	Extravasation: 4.5% (8/177)	NA	NA	NA	NA	NA	Phentolamine was used in 1 case; conservative management for others

© 2026 ZhangJian SJ et al. *JAMA Network Open*.

Author, year	Total number of AE	Age (years)[a]	Male (%)	Vasopressor dose[a]	Vasopressor duration[a]	Gauge, No. (%)	Location, No. (%)	AE detail per vasopressor medication						Intervention for AE
								Norepinephrine	Dopamine	Epinephrine	Phenylephrine	Vasopressin	Metaraminol	
							(37.5)							
Padmanaban et al, [33] 2020	1	NR	NR	Norepinephrine mean dose of 6.5 mcg/min and a maximum dose of 9 mcg/min	52h	20G	Antecubital: 1 (100)	Extravasation: 0.8% (1/118)	0% (0/2)	0% (0/6)	NA	0% (0/29)	NA	Conservative treatment; no antidote used
Pancaro et al, [34] 2020	5	66.2 (±18.4)	2 (40)	Norepinephrine dose range: 0.02-0.05 mcg/kg/min	Norepinephrine: 20min (20–25)	18G (18-18)	ACF: 3 (60); Hand: 1 (20); Lower extremities: 1 (20)	Extravasation: 0.03% (5/14385)	NA	NA	NA	NA	NA	Stop infusion and monitor
Petros et al, [35] 2025	3	87; 87; 70	1 (33)	NR	All > 5 days	20G: 2 (66.6); 18G: 1 (33.4)	Hand: 1 (33.4); Forearm: 2 (66.6)	Extravasation: 1.2% (3/250)	0	0	NA	NA	NA	No active management needed
Powell et al, [36] 2023	0	NA	NA	NA	NA	NA	NA	0	NA	NA	NA	NA	NA	NA
Prasanna et al, [37] 2021	4	NR	NR	NR	NR	Midline catheter: 4 Fr 20cm	NR	NR	NA	NR	NR	NR	NA	Immediate drip discontinuation
Putland et al, [38] 2006	7	NR	NR	NR	NR	NR	NR	NR	NR	Local tissue pallor or	NR	NR	NR	NR

© 2026 ZhangJian SJ et al. *JAMA Network Open*.

Author, year	Total number of AE	Age (years)[a]	Male (%)	Vasopressor dose[a]	Vasopressor duration[a]	Gauge, No. (%)	Location, No. (%)	AE detail per vasopressor medication						Intervention for AE
								Norepinephrine	Dopamine	Epinephrine	Phenylephrine	Vasopressin	Metaraminol	
										mottling: 5% (7/220)				
Ramanan et al, [39] 2025	7	NR	NR	NR	NR	NR	NR	NR	NR	NR	NR	NR	NR	NR
Ruchti et al, [40] 2021	2	NR	NR	NR	NR	18G or larger	NR	Extravasation: 4% (2/50)	NA	NA	NA	NA	0	No treatment required
Sardaneh et al, [41] 2021	0	NA	NA	NA	NA	NA	NA	NA	NA	NA	NA	NA	NA	NA
Schmucki, et al, [42] 2025	252	NR	NR	NR	NR	NR	NR	Reddening: 2.5% (39/1561); Hematoma: 13.3% (208/1561); Edema: 0.3% (5/1561)	NA	NA	NA	NA	NA	Local treatment only
Shyu et al, [43] 2025	31	NR	NR	NR	NR	NR	NR	NR	NR	NR	NR	NR	NR	30 require phentolamine administration, 1 requiring fasciotomy
Spiegel et al, [44] 2020	1	NR	NR	NR	NR	NR	NR	Extravasation: 0.8% (1/119)	NR	NR	NR	NR	NR	Phentolamine was used with conservative treatment
Stolz et al, [45] 2022	72	68.4 (52.9-75.7)	NR	NR	NR	NR	NR	NR	NA	NR	NR	NA	NR	NR

© 2026 ZhangJian SJ et al. *JAMA Network Open*.

Author, year	Total number of AE	Age (years)[a]	Male (%)	Vasopressor dose[a]	Vasopressor duration[a]	Gauge, No. (%)	Location, No. (%)	AE detail per vasopressor medication						Intervention for AE
								Norepinephrine	Dopamine	Epinephrine	Phenylephrine	Vasopressin	Metaraminol	
Vitharana et al,[46] 2023	20	NR	NR	NR	NR	NR	NR	Pain at site: 30.7% (16/52); Extravasation: 7.6% (4/52); Swelling: 11.5% (6/52)	NA	NA	NA	NA	NA	Replace IV site
Yasuda et al,[47] 2022	19	NR	NR	NR	NR	NR	NR	Phlebitis: 21.6% (19/88)	NA	NA	NA	NA	NA	NR
Yerke et al,[48] 2024	35	67 (61-74)	NR	Norepinephrine Max: 13 mcg/min (7-15)	13.8h (4.0-29.5)	NR	NR	Extravasation: 5.5% (35/635)	NA	NA	NA	NA	NA	Subcutaneous phentolamine and nitroglycerin paste
Zichichi et al,[49] 2024	11	NR	NR	Norepinephrine Max: 0.1 mcg/kg/min (0.07-0.12) Phenylephrine Max: 2 mcg/kg/min (1.25-4)	NR	16G: 0 (0); 18G: 5 (45.5); 20G: 3 (27.3); Others: 2 (18.2); No documentation: 1 (9)	ACF: 2 (18.2); Upper extremity: 4 (36.4); Forearm: 2 (18.2); Metacarpal: 2 (18.2); Others: 1 (9)	Extravasation: 5.6% (8/142)	NA	0	Extravasation: 6.3% (3/48)	NA	NA	Warm or cold compress: 10; Topical nitroglycerin: 3; Terbutaline: 3

Abbreviations: AE, adverse event; ACF, antecubital fossa; IV, intravenous; NA, not applicable; NR, not recorded; PIV, peripheral intravenous; PIVC, peripheral intravenous catheter

[a] Data presented as "mean (± standard deviation)" or "median (interquartile)" or "median (range)"

© 2026 ZhangJian SJ et al. *JAMA Network Open*.

eTable 6. Summary of subgroup analysis of minor adverse events.

Subgroup	Studies (events/catheters)	Pooled incidence proportion of minor AEs (95% CI)	I^2(%)	Subgroup difference *P* value
Overall	48 (735/31212)	2.34% (1.47-3.72%)	95	
Publishing year				0.05[a]
＞ 2021	28 (652/13608)	3.40% (1.93-5.92%)	96	
≤ 2021	20 (83/17604)	1.36% (0.67-2.77%)	84	
Study setting[b]				0.41
ED	9 (23/1319)	1.65% (0.86-3.15%)	25	
ICU	25 (347/10946)	3.30% (2.13-5.10%)	95	
Mixed	6 (23/1341)	0.98% (0.12-7.65%)	71	
OR	4 (283/16980)	1.89% (0.17-18.0%)	99	
Ward	4 (59/626)	2.39% (0.08-41.9%)	91	
Study design				<0.01[a]
Prospective observational	14 (384/4537)	4.52% (2.35-8.52%)	94	
Retrospective observational	30 (340/26577)	1.47% (0.78-2.79%)	95	
Randomized controlled trial	4 (11/98)	11.1% (5.16-22.3%)	0	
Duration of infusion[c] (hours)				0.28
≥ 24	13 (194/6436)	3.81% (1.66-8.52%)	97	
＜ 24	26 (476/8880)	2.23% (1.34-3.69%)	92	
PIV gauge (G)				0.23
≥ 22 (smaller)	8 (4/57)	7.02% (2.66-17.3%)	0	
＜ 22 (larger)	16 (101/2020)	3.63% (2.40-5.45%)	54	
PIV location				0.57
Including and above antecubital fossa	17 (89/2119)	2.15% (1.09-4.20%)	58	
Below antecubital fossa	15 (71/1898)	2.88% (1.76-4.69%)	2	
Others	9 (1/86)	1.16% (0.16-7.79%)	0	
Protocol use				0.68
With protocol	30 (564/27648)	2.49% (1.45-4.24%)	96	
Without protocol	18 (171/3564)	1.99% (0.78-4.96%)	93	
Catheter type				0.89
Midline	4 (55/1126)	2.62% (0.89-7.50%)	83	
Short PIV	43 (677/29596)	2.41% (1.44-3.98%)	96	

Abbreviations: AEs, adverse events; CI, confidence interval; ED, emergency department; G, gauge; ICU, intensive care unit; OR, operating room; PIV, peripheral intravenous.

[a] $P < 0.05$

[b] One study conducted in the intermediate care unit were categorized as a ward-based study.

[c] Duration refers to the mean or median length of peripheral vasopressor administration as reported in the included studies.

© 2026 ZhangJian SJ et al. *JAMA Network Open*.

eTable 7. Summary of subgroup analysis of central venous catheter avoidance proportion.

**Subgroup**	**Studies (events/catheters)**	**Pooled proportion of CVC avoidance (95% CI)**	I^2**(%)**	**Subgroup difference *P* value**
Overall	38 (7459/15371)	59.7% (46.4-71.7%)	99	
Publishing year				0.70
> 2021	23 (5673/12522)	57.2% (42.1-71.1%)	99	
≤ 2021	15 (1786/2849)	62.8% (38.4-82.1%)	99	
Study setting[b]				<0.01[a]
ED	7 (505/980)	40.4% (13.1-75.3%)	96	
ICU	20 (5110/11640)	54.8% (43.2-65.9%)	99	
Mixed	5 (342/1091)	35.6% (11.0-71.4%)	98	
OR	2 (981/1034)	94.9% (93.4-96.1%)	31	
Ward	4 (521/626)	95.4% (80.2-99.1%)	78	
Study design				0.09
Prospective observational	8 (1606/2124)	77.9% (60.7-89.0%)	98	
Retrospective observational	27 (5822/13160)	56.9% (42.2-70.4%)	99	
Randomized controlled trial	3 (31/87)	15.0% (0.30-91.2%)	93	
Duration of infusion (hours)[c]				0.51
≥ 24	11 (3305/7639)	55.2% (31.8-76.4%)	99	
< 24	23 (3826/7079)	64.7% (48.6-78.0%)	98	
Protocol use				0.72
With protocol	26 (6395/13191)	61.3% (46.7-74.0%)	99	
Without protocol	12 (1064/2180)	55.5% (28.2-79.8%)	98	
Catheter type				<0.01[a]
Midline	2 (591/720)	83.8% (75.9-89.4%)	90	
Short PIV	35 (6608/14161)	58.1% (43.8-71.2%)	99	

Abbreviations: CVC, central venous catheter; CI, confidence interval; ED, emergency department; ICU, intensive care unit; OR, operating room; PIV, peripheral intravenous.

[a] $P < 0.05$

[b] One study conducted in the intermediate care unit were categorized as a ward-based study.

[c] Duration refers to the mean or median length of peripheral vasopressor administration as reported in the included studies.

© 2026 ZhangJian SJ et al. *JAMA Network Open*.

eTable 8. Sensitivity analyses of the pooled incidence proportion of minor adverse events: excluding high-risk-of-bias studies and using the leave-one-out method.

**Author, year**	**Pooled incidence proportion of minor adverse events (95% CI)**	I^2
Overall	2.34% (1.47-3.72%)	95%
Excluding studies with high risk of bias	2.06% (1.30-3.25%)	95%
Excluding Andrews 2017	2.39% (1.49-3.79%)	95%
Excluding Asher 2023	2.28% (1.41-3.66%)	95%
Excluding Aykanat 2022	2.27% (1.41-3.64%)	95%
Excluding Ballieu 2021	2.27% (1.41-3.65%)	95%
Excluding Bima 2022	2.40% (1.50-3.81%)	95%
Excluding Cape 2022	2.32% (1.44-3.73%)	95%
Excluding Cardenas-Garcia 2015	2.32% (1.44-3.74%)	95%
Excluding Christensen 2024	2.33% (1.44-3.75%)	95%
Excluding Dansereau 2024	2.30% (1.42-3.71%)	95%
Excluding Datar 2018	2.31% (1.43-3.72%)	95%
Excluding Delaney 2019	2.53% (1.61-3.95%)	95%
Excluding Delgado 2016	2.32% (1.44-3.71%)	95%
Excluding Fabick 2023	2.32% (1.43-3.74%)	95%
Excluding Feng 2021	2.29% (1.42-3.68%)	95%
Excluding Gershengorn 2023	2.29% (1.42-3.69%)	95%
Excluding Groetzinger 2022	2.32% (1.43-3.72%)	95%
Excluding Hallengren 2017	2.44% (1.53-3.86%)	95%
Excluding Han 2024	2.35% (1.45-3.77%)	95%
Excluding He 2022	2.29% (1.42-3.69%)	95%
Excluding Johnson 1977	2.30% (1.43-3.68%)	95%
Excluding Karlsson 2024	2.26% (1.40-3.63%)	95%
Excluding Kilian 2022	2.41% (1.51-3.82%)	95%
Excluding Lewis 2019	2.30% (1.42-3.70%)	95%
Excluding Marques 2022	2.33% (1.44-3.74%)	95%
Excluding Marti 2022	2.34% (1.45-3.76%)	95%
Excluding McCurry 2025	2.44% (1.53-3.86%)	95%
Excluding Medlej 2018	2.29% (1.42-3.69%)	95%
Excluding Messina 2021	2.31% (1.43-3.71%)	95%
Excluding Munroe 2024	2.53% (1.61-3.95%)	95%
Excluding Munroe 2025	2.42% (1.51-3.86%)	95%
Excluding Nguyen 2021	2.34% (1.45-3.76%)	95%
Excluding Padmanaban 2020	2.40% (1.49-3.83%)	95%
Excluding Pancaro 2020	2.73% (1.80-4.12%)	94%
Excluding Petros 2025	2.38% (1.47-3.81%)	95%

© 2026 ZhangJian SJ et al. *JAMA Network Open*.

Excluding Powell 2023	2.45% (1.54-3.87%)	95%
Excluding Prasanna 2021	2.40% (1.50-3.84%)	95%
Excluding Putland 2006	2.31% (1.43-3.73%)	95%
Excluding Ramanan 2025	2.24% (1.40-3.56%)	95%
Excluding Ruchti 2021	2.35% (1.46-3.77%)	95%
Excluding Sardaneh 2021	2.44% (1.53-3.86%)	95%
Excluding Schmucki 2025	2.23% (1.39-3.55%)	94%
Excluding Shyu 2025	2.40% (1.49-3.85%)	94%
Excluding Spiegel 2020	2.40% (1.49-3.83%)	95%
Excluding Stolz 2022	2.21% (1.40-3.45%)	94%
Excluding Vitharana 2023	2.21% (1.40-3.45%)	95%
Excluding Yasuda 2022	2.22% (1.39-3.53%)	95%
Excluding Yerke 2024	2.28% (1.41-3.67%)	95%
Excluding Zichichi 2024	2.28% (1.41-3.67%)	95%

Abbreviations: CI, confidence interval

© 2026 ZhangJian SJ et al. *JAMA Network Open*.

eTable 9. Sensitivity analyses of the pooled proportion of central venous catheter avoidance: excluding high risk of bias studies and using the leave-one-out method.

Author, year	Pooled incidence proportion of minor adverse events (95% CI)	I^2
Overall	59.7% (46.4-71.7%)	99%
Excluding studies with high risk of bias	60.0% (46.8-71.9%)	95%
Excluding Andrews 2017	61.77% (49.12-73.01%)	99%
Excluding Aykanat 2022	58.55% (45.10-70.83%)	99%
Excluding Ballieu 2021	60.69% (47.19-72.73%)	99%
Excluding Bima 2022	57.72% (44.80-69.66%)	99%
Excluding Cape 2022	60.43% (46.84-72.58%)	99%
Excluding Cardenas-Garcia 2015	58.68% (45.13-71.03%)	98%
Excluding Christensen 2024	57.94% (44.76-70.08%)	98%
Excluding Dansereau 2024	60.31% (46.67-72.51%)	99%
Excluding Datar 2018	59.06% (45.41-71.45%)	99%
Excluding Delaney 2019	61.61% (48.71-73.07%)	99%
Excluding Fabick 2023	60.29% (46.65-72.50%)	99%
Excluding Feng 2021	60.38% (46.77-72.55%)	99%
Excluding Gandotra 2023	59.90% (46.20-72.22%)	99%
Excluding Groetzinger 2022	59.97% (46.27-72.26%)	99%
Excluding Hallengren 2017	57.41% (44.68-69.24%)	99%
Excluding Han 2024	59.03% (45.38-71.41%)	99%
Excluding Karlsson 2024	59.09% (45.43-71.48%)	99%
Excluding Kilian 2022	61.21% (48.03-72.94%)	99%
Excluding Lewis 2019	59.88% (46.17-72.20%)	99%
Excluding Marques 2022	58.57% (45.09-70.88%)	99%
Excluding Marti 2022	60.40% (46.79-72.56%)	99%
Excluding McCurry 2025	59.49% (45.78-71.86%)	99%
Excluding Medlej 2018	59.19% (45.52-71.57%)	99%
Excluding Messina 2021	58.38% (44.97-70.66%)	99%
Excluding Munroe 2024	60.43% (46.84-72.59%)	99%
Excluding Munroe 2025	59.90% (46.20-72.22%)	99%
Excluding Nguyen 2021	61.05% (47.73-72.90%)	99%
Excluding Padmanaban 2020	59.47% (45.76-71.85%)	99%
Excluding Powell 2023	60.34% (46.72-72.53%)	99%
Excluding Prasanna 2021	58.60% (45.09-70.93%)	99%
Excluding Ramanan 2025	61.32% (48.22-72.97%)	99%
Excluding Ruchti 2021	59.04% (45.40-71.42%)	99%
Excluding Sardaneh 2021	58.80% (45.22-71.15%)	99%
Excluding Shyu 2025	60.72% (47.23-72.75%)	98%

© 2026 ZhangJian SJ et al. *JAMA Network Open*.

Excluding Stolz 2022	60.89% (47.49-72.83%)	99%
Excluding Vitharana 2023	58.34% (44.97-70.58%)	99%
Excluding Yerke 2024	60.01% (46.32-72.30%)	99%
Excluding Zichichi 2024	59.74% (46.02-72.08%)	99%

Abbreviations: CI, confidence interval

© 2026 ZhangJian SJ et al. *JAMA Network Open*.

eFigure 1. Forest plot showing the pooled incidence proportions of minor adverse events associated with individual vasopressor agents. 95% CI indicates 95% confidence interval.

Study	Events	Catheters	Proportion	95% CI
**A Vasopressor type = Norepinephrine**				
Aykanat et al, 2022	3	30	0.100	[0.021; 0.265]
Bima et al, 2022	0	23	0.000	[0.000; 0.148]
Cape et al, 2022	3	92	0.033	[0.007; 0.092]
Cardenas−Garcia et al, 2015	16	506	0.032	[0.018; 0.051]
Christensen et al, 2024	110	1004	0.110	[0.091; 0.131]
Dansereau et al, 2024	11	226	0.049	[0.025; 0.085]
Delaney et al, 2019	0	293	0.000	[0.000; 0.013]
Fabick et al, 2023	27	1340	0.020	[0.013; 0.029]
Feng et al, 2021	6	116	0.052	[0.019; 0.109]
Groetzinger et al, 2022	3	87	0.034	[0.007; 0.097]
Hallengren et al, 2017	0	79	0.000	[0.000; 0.046]
He et al, 2022	27	640	0.042	[0.028; 0.061]
Karlsson et al, 2024	39	472	0.083	[0.059; 0.111]
Kilian et al, 2022	0	29	0.000	[0.000; 0.119]
Lewis et al, 2019	4	146	0.027	[0.008; 0.069]
Marti et al, 2022	2	67	0.030	[0.004; 0.104]
Medlej et al, 2018	3	50	0.060	[0.013; 0.165]
Messina et al, 2021	5	127	0.039	[0.013; 0.089]
Munroe et al, 2024	0	337	0.000	[0.000; 0.011]
Nguyen et al, 2021	8	177	0.045	[0.020; 0.087]
Padmanaban et al, 2020	1	122	0.008	[0.000; 0.045]
Pancaro et al, 2020	5	14385	0.000	[0.000; 0.001]
Petros et al, 2025	3	144	0.021	[0.004; 0.060]
Powell et al, 2023	0	98	0.000	[0.000; 0.037]
Ruchti et al, 2021	2	50	0.040	[0.005; 0.137]
Schmucki et al, 2025	252	1561	0.161	[0.144; 0.181]
Vitharana et al, 2023	20	52	0.385	[0.253; 0.530]
Yasuda et al, 2022	19	88	0.216	[0.135; 0.316]
Yerke et al, 2024	35	635	0.055	[0.039; 0.076]
Zichichi et al, 2024	8	142	0.056	[0.025; 0.108]
Total		23118	0.026	[0.014; 0.047]
Heterogeneity: $\chi^2_{29} = 471.63$ ($P < .001$), $I^2 = 93.9\%$				
**B Vasopressor type = Epinephrine**				
Dansereau et al, 2024	0	9	0.000	[0.000; 0.336]
Delaney et al, 2019	0	38	0.000	[0.000; 0.093]
Fabick et al, 2023	0	107	0.000	[0.000; 0.034]
Kilian et al, 2022	0	2	0.000	[0.000; 0.842]
Lewis et al, 2019	0	2	0.000	[0.000; 0.842]
Marti et al, 2022	0	3	0.000	[0.000; 0.708]
Munroe et al, 2024	0	28	0.000	[0.000; 0.123]
Padmanaban et al, 2020	0	122	0.000	[0.000; 0.030]
Petros et al, 2025	0	104	0.000	[0.000; 0.035]
Putland et al, 2006	7	220	0.032	[0.013; 0.064]
Zichichi et al, 2024	0	8	0.000	[0.000; 0.369]
Total		643	0.000	[0.000; 0.246]
Heterogeneity: $\chi^2_{10} = 0$ ($P > .99$), $I^2 = 0\%$				
**C Vasopressor type = Phenylephrine**				
Ballieu et al, 2021	9	125	0.072	[0.033; 0.132]
Cardenas−Garcia et al, 2015	0	176	0.000	[0.000; 0.021]
Dansereau et al, 2024	3	139	0.022	[0.004; 0.062]
Datar et al, 2018	9	277	0.032	[0.015; 0.061]
Delgado et al, 2016	2	20	0.100	[0.012; 0.317]
Fabick et al, 2023	19	815	0.023	[0.014; 0.036]
Kilian et al, 2022	0	3	0.000	[0.000; 0.708]
Lewis et al, 2019	4	73	0.055	[0.015; 0.134]
Marti et al, 2022	1	58	0.017	[0.000; 0.092]
Munroe et al, 2024	0	22	0.000	[0.000; 0.154]
Zichichi et al, 2024	3	48	0.062	[0.013; 0.172]
Total		1756	0.029	[0.018; 0.048]
Heterogeneity: $\chi^2_{10} = 13.47$ ($P = .20$), $I^2 = 25.8\%$				
**D Vasopressor type = Dopamine**				
Andrews et al, 2017	0	17	0.000	[0.000; 0.195]
Cardenas−Garcia et al, 2015	3	101	0.030	[0.006; 0.084]
Fabick et al, 2023	4	98	0.041	[0.011; 0.101]
Lewis et al, 2019	0	2	0.000	[0.000; 0.842]
Medlej et al, 2018	0	5	0.000	[0.000; 0.522]
Munroe et al, 2024	0	13	0.000	[0.000; 0.247]
Padmanaban et al, 2020	0	122	0.000	[0.000; 0.030]
Petros et al, 2025	0	17	0.000	[0.000; 0.195]
Total		375	0.014	[0.003; 0.059]
Heterogeneity: $\chi^2_{7} = 0.18$ ($P > .99$), $I^2 = 0\%$				
**E Vasopressor type = Vasopressin**				
Dansereau et al, 2024	0	5	0.000	[0.000; 0.522]
Fabick et al, 2023	1	217	0.005	[0.000; 0.025]
Johnson et al, 1977	1	11	0.091	[0.002; 0.413]
Lewis et al, 2019	0	4	0.000	[0.000; 0.602]
Marti et al, 2022	0	1	0.000	[0.000; 0.975]
McCurry et al, 2026	0	80	0.000	[0.000; 0.045]
Padmanaban et al, 2020	0	122	0.000	[0.000; 0.030]
Total		440	0.005	[0.001; 0.018]
Heterogeneity: $\chi^2_{6} = 4.49$ ($P = .61$), $I^2 = 0\%$				
**F Vasopressor type = Metaraminol**				
Delaney et al, 2019	0	58	0.000	[0.000; 0.062]
Han et al, 2024	5	273	0.018	[0.006; 0.042]
Ruchti et al, 2021	0	50	0.000	[0.000; 0.071]
Sardaneh et al, 2021	0	81	0.000	[0.000; 0.045]
Total		462	0.009	[0.001; 0.115]
Heterogeneity: $\chi^2_{3} = 0$ ($P > .99$), $I^2 = 0\%$				

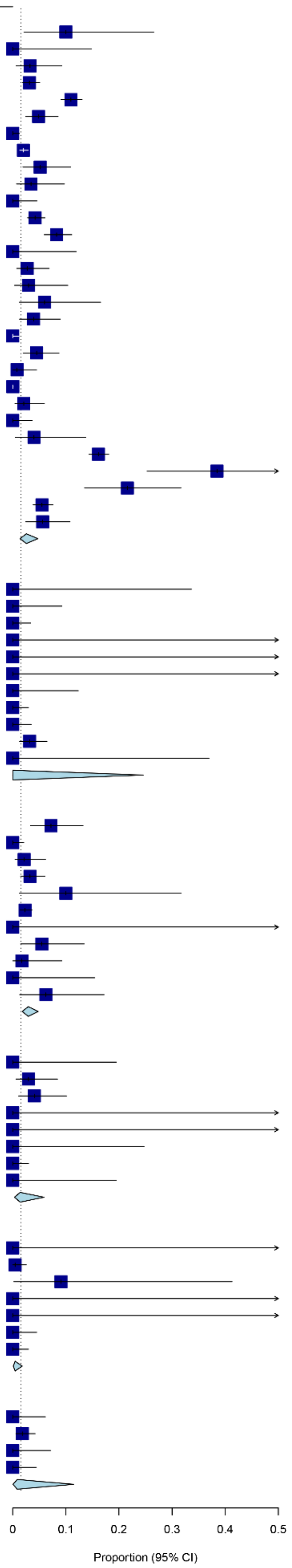

© 2026 ZhangJian SJ et al. *JAMA Network Open*.

eFigure 2. Forest plot of pooled incidence proportion of minor adverse events across all vasopressor agents. 95% CI indicates 95% confidence interval.

Study	Events	Catheters	Proportion	95% CI
Andrews et al, 2017	0	17	0.000	[0.000; 0.195]
Asher et al, 2023	7	108	0.065	[0.026; 0.129]
Aykanat et al, 2022	3	30	0.100	[0.021; 0.265]
Ballieu et al, 2021	9	125	0.072	[0.033; 0.132]
Bima et al, 2022	0	23	0.000	[0.000; 0.148]
Cape et al, 2022	3	92	0.033	[0.007; 0.092]
Cardenas−Garcia et al, 2015	19	734	0.026	[0.016; 0.040]
Christensen et al, 2024	23	1004	0.023	[0.015; 0.034]
Dansereau et al, 2024	14	382	0.037	[0.020; 0.061]
Datar et al, 2018	9	277	0.032	[0.015; 0.061]
Delaney et al, 2019	0	389	0.000	[0.000; 0.009]
Delgado et al, 2016	1	20	0.050	[0.001; 0.249]
Fabick et al, 2023	58	2163	0.027	[0.020; 0.035]
Feng et al, 2021	6	116	0.052	[0.019; 0.109]
Gershengorn et al, 2023	13	287	0.045	[0.024; 0.076]
Groetzinger et al, 2022	3	87	0.034	[0.007; 0.097]
Hallengren et al, 2017	0	79	0.000	[0.000; 0.046]
Han et al, 2024	5	273	0.018	[0.006; 0.042]
He et al, 2022	27	640	0.042	[0.028; 0.061]
Johnson et al, 1977	1	11	0.091	[0.002; 0.413]
Karlsson et al, 2024	39	472	0.083	[0.059; 0.111]
Kilian et al, 2022	0	34	0.000	[0.000; 0.103]
Lewis et al, 2019	8	202	0.040	[0.017; 0.077]
Marques et al, 2022	2	64	0.031	[0.004; 0.108]
Marti et al, 2022	3	129	0.023	[0.005; 0.066]
McCurry et al, 2025	0	80	0.000	[0.000; 0.045]
Medlej et al, 2018	3	55	0.055	[0.011; 0.151]
Messina et al, 2021	5	127	0.039	[0.013; 0.089]
Munroe et al, 2024	0	400	0.000	[0.000; 0.009]
Munroe et al, 2025	3	490	0.006	[0.001; 0.018]
Nguyen et al, 2021	4	177	0.023	[0.006; 0.057]
Padmanaban et al, 2020	1	122	0.008	[0.000; 0.045]
Pancaro et al, 2020	5	14385	0.000	[0.000; 0.001]
Petros et al, 2025	3	250	0.012	[0.002; 0.035]
Powell et al, 2023	0	98	0.000	[0.000; 0.037]
Prasanna et al, 2021	2	248	0.008	[0.001; 0.029]
Putland et al, 2006	7	220	0.032	[0.013; 0.064]
Ramanan et al, 2025	7	40	0.175	[0.073; 0.328]
Ruchti et al, 2021	2	100	0.020	[0.002; 0.070]
Sardaneh et al, 2021	0	81	0.000	[0.000; 0.045]
Schmucki et al, 2025	252	1561	0.161	[0.144; 0.181]
Shyu et al, 2025	30	3734	0.008	[0.005; 0.011]
Spiegel et al, 2020	1	119	0.008	[0.000; 0.046]
Stolz et al, 2022	72	194	0.371	[0.303; 0.443]
Vitharana et al, 2023	20	52	0.385	[0.253; 0.530]
Yasuda et al, 2022	19	88	0.216	[0.135; 0.316]
Yerke et al, 2024	35	635	0.055	[0.039; 0.076]
Zichichi et al, 2024	11	198	0.056	[0.028; 0.097]
Total		31212	0.023	[0.015; 0.037]

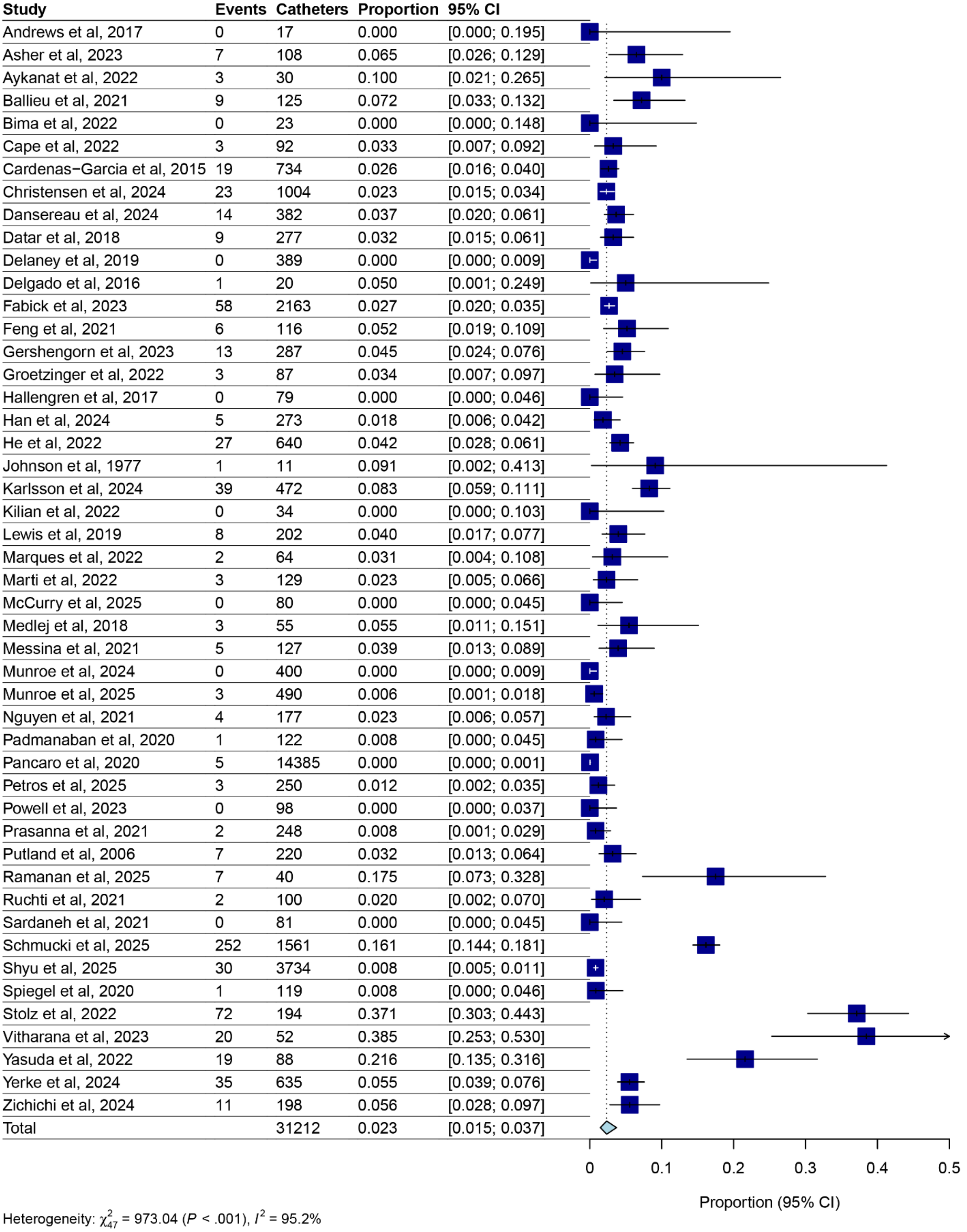

Heterogeneity: χ^2_{47} = 973.04 (P < .001), I^2 = 95.2%

© 2026 ZhangJian SJ et al. *JAMA Network Open*.

eFigure 3. Doi plot visualization and the Luis Furuya-Kanamori (LFK) asymmetry index for publication bias assessment.

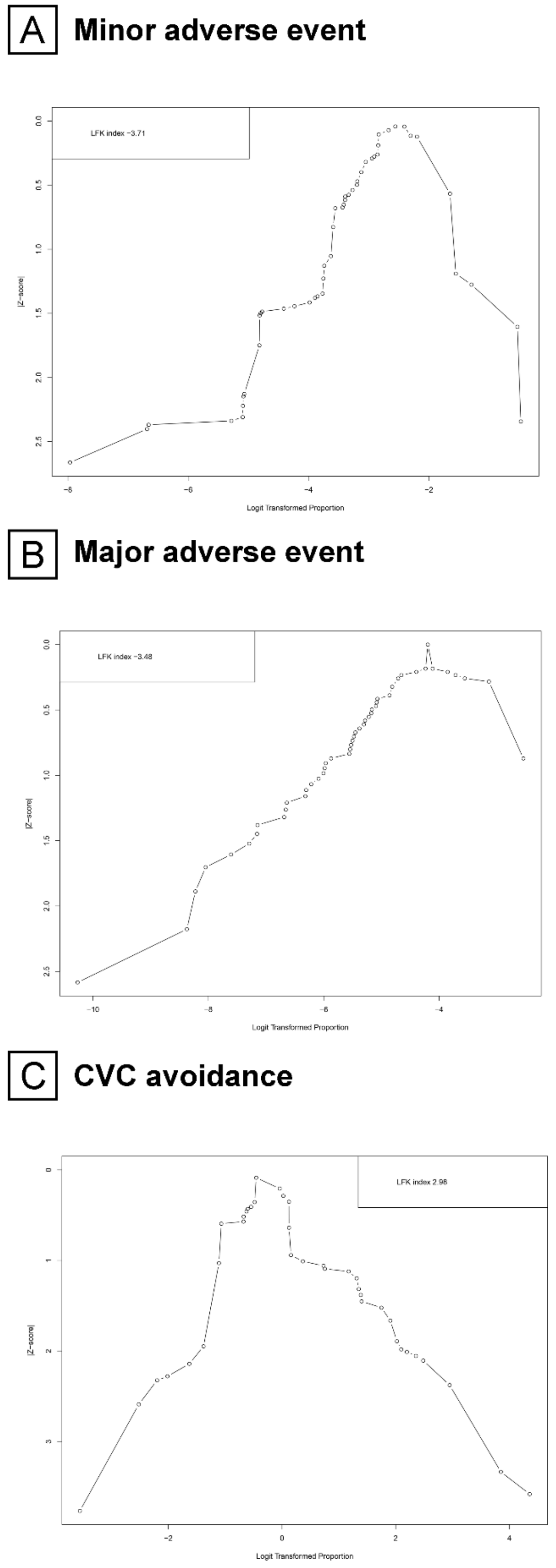

© 2026 ZhangJian SJ et al. *JAMA Network Open*.

eReferences

1. Andrews B, Semler MW, Muchemwa L, et al. Effect of an Early Resuscitation Protocol on In-hospital Mortality Among Adults With Sepsis and Hypotension: A Randomized Clinical Trial. *Jama*. Oct 3 2017;318(13):1233–1240. doi:10.1001/jama.2017.10913
2. Asher E, Karameh H, Nassar H, et al. Safety and Outcomes of Peripherally Administered Vasopressor Infusion in Patients Admitted with Shock to an Intensive Cardiac Care Unit-A Single-Center Prospective Study. *J Clin Med*. Sep 3 2023;12(17)doi:10.3390/jcm12175734
3. Aykanat VM, Myles PS, Weinberg L, Burrell A, Bellomo R. Low-Concentration Norepinephrine Infusion for Major Surgery: a Safety and Feasibility Pilot Randomized Controlled Trial. Journal article. *Anesthesia and analgesia*. 2022;134(2):410–418. doi:10.1213/ANE.0000000000005811
4. Ballieu P, Besharatian Y, Ansari S. Safety and Feasibility of Phenylephrine Administration Through a Peripheral Intravenous Catheter in a Neurocritical Care Unit. *J Intensive Care Med*. Jan 2021;36(1):101–106. doi:10.1177/0885066619887111
5. Bima P, Orlotti C, Smart OG, et al. Norepinephrine may improve survival of septic shock patients in a low-resource setting: a proof-of-concept study on feasibility and efficacy outside the Intensive Care Unit. *Pathog Glob Health*. Sep 2022;116(6):389–394. doi:10.1080/20477724.2022.2038051
6. Cape KM, Jones LG, Weber ML, Elefritz JL. Implementation of a Protocol for Peripheral Intravenous Norepinephrine: Does It Save Central Line Insertion, Is It Safe? *J Pharm Pract*. Jun 2022;35(3):347–351. doi:10.1177/0897190020977712
7. Cardenas-Garcia J, Schaub KF, Belchikov YG, Narasimhan M, Koenig SJ, Mayo PH. Safety of peripheral intravenous administration of vasoactive medication. *J Hosp Med*. Sep 2015;10(9):581–5. doi:10.1002/jhm.2394
8. Christensen J, Andersson E, Sjöberg F, et al. Adverse Events of Peripherally Administered Norepinephrine During Surgery: A Prospective Multicenter Study. *Anesth Analg*. Jun 1 2024;138(6):1242–1248. doi:10.1213/ane.0000000000006806
9. Dansereau AC, Marti KE, Mah JW, Pugliese NM. Evaluation of the safety and efficacy of peripheral vasopressors to decrease central line placement and associated bloodstream infections. *J Infect Prev*. Sep 2024;25(5):153–160. doi:10.1177/17571774241245437
10. Datar S, Gutierrez E, Schertz A, Vachharajani V. Safety of Phenylephrine Infusion Through Peripheral Intravenous Catheter in the Neurological Intensive Care Unit. *J Intensive Care Med*. Oct 2018;33(10):589–592. doi:10.1177/0885066617712214
11. Delaney A, Finnis M, Bellomo R, et al. Initiation of vasopressor infusions via peripheral versus central access in patients with early septic shock: A retrospective cohort study. *Emerg Med Australas*. Apr 2020;32(2):210–219. doi:10.1111/1742-6723.13394
12. Delgado T, Wolfe B, Davis G, Ansari S. Safety of peripheral administration of phenylephrine in a neurologic intensive care unit: A pilot study. *J Crit Care*. Aug 2016;34:107–10. doi:10.1016/j.jcrc.2016.04.004
13. Fabick AC, Hawn JM, Barwick KW, Weeda ER, Goodwin AJ, Bell CM. Comparison of extravasation events related to the peripheral administration of vasopressors prior to and following

© 2026 ZhangJian SJ et al. *JAMA Network Open*.

implementation of an institutional protocol. Article. *JACCP Journal of the American College of Clinical Pharmacy*. 2023;6(7):709–717. doi:10.1002/jac5.1844

14. Feng F, Yang W, Zhang Z, Mu C, Li M, Chen Y. [Safety of administration of norepinephrine through peripheral vein line in patients with septic shock]. *Zhonghua Wei Zhong Bing Ji Jiu Yi Xue*. Mar 2021;33(3):276–280. doi:10.3760/cma.j.cn121430-20200716-00528

15. Gandotra S, Wunsch H, Bosch NA, Walkey AJ, Teja B. Reducing Central Venous Catheter Use through Adoption of Guidelines for Peripheral Catheter-based Vasopressor Delivery. *Ann Am Thorac Soc*. Aug 2023;20(8):1219–1223. doi:10.1513/AnnalsATS.202212-1060RL

16. Gershengorn HB, Basu T, Horowitz JK, et al. The Association of Vasopressor Administration through a Midline Catheter with Catheter-related Complications. *Ann Am Thorac Soc*. Jul 2023;20(7):1003–1011. doi:10.1513/AnnalsATS.202209-814OC

17. Groetzinger LM, Williams J, Svec S, Donahoe MP, Lamberty PE, Barbash IJ. Peripherally Infused Norepinephrine to Avoid Central Venous Catheter Placement in a Medical Intensive Care Unit: A Pilot Study. *Ann Pharmacother*. Jul 2022;56(7):773–781. doi:10.1177/10600280211053318

18. Hallengren M, Åstrand P, Eksborg S, Barle H, Frostell C. Septic shock and the use of norepinephrine in an intermediate care unit: Mortality and adverse events. *PLoS One*. 2017;12(8):e0183073. doi:10.1371/journal.pone.0183073

19. Han P, Zhou Y. Safety and efficacy of peripheral metaraminol infusion in patients with neurological conditions: a single-center retrospective observational study. *Front Neurol*. 2024;15:1398827. doi:10.3389/fneur.2024.1398827

20. He L WD, Yin L. Risk factor analysis and early-warning management of safety in peripheral intravenous norepinephrine infusion for septic shock patients. *Chinese General Practice Nursing*. 2022;20(10):1418–21. doi:10.12104/j.issn.1674-4748.2022.10.035

21. Johnson WC, Widrich WC, Ansell JE, Robbins AH, Nabseth DC. Control of bleeding varices by vasopressin: a prospective randomized study. *Ann Surg*. Sep 1977;186(3):369–76. doi:10.1097/00000658-197709000-00015

22. Karlsson H, Afrasiabi A, Ohlsson M, Månsson V, Hartman H, Torisson G. Treating shock with norepinephrine administered in midline catheters in an intermediary care unit: a retrospective cohort study. *BMJ Open*. Dec 30 2024;14(12):e091311. doi:10.1136/bmjopen-2024-091311

23. Kilian S, Surrey A, McCarron W, Mueller K, Wessman BT. Vasopressor Administration via Peripheral Intravenous Access for Emergency Department Stabilization in Septic Shock Patients. *Indian J Crit Care Med*. Jul 2022;26(7):811–815. doi:10.5005/jp-journals-10071-24243

24. Lewis T, Merchan C, Altshuler D, Papadopoulos J. Safety of the Peripheral Administration of Vasopressor Agents. *J Intensive Care Med*. Jan 2019;34(1):26–33. doi:10.1177/0885066616686035

25. Marques CG, Mwemerashyaka L, Martin K, et al. Utilisation of peripheral vasopressor medications and extravasation events among critically ill patients in Rwanda: A prospective cohort study. *Afr J Emerg Med*. Jun 2022;12(2):154–159. doi:10.1016/j.afjem.2022.03.006

26. Marti K, Hartley C, Sweeney E, Mah J, Pugliese N. Evaluation of the safety of a novel peripheral vasopressor pilot program and the impact on central line placement in medical and surgical intensive care units. *Am J Health Syst Pharm*. Aug 19 2022;79(Suppl 3):S79–s85. doi:10.1093/ajhp/zxac144
© 2026 ZhangJian SJ et al. *JAMA Network Open*.

27. McCurry K, DeWitt K, Upchurch CP, Wren RN. Peripherally administered vasopressin initiated in the emergency department. *Journal of Critical Care*. 2026/04/01/ 2026;92:155363. doi:https://doi.org/10.1016/j.jcrc.2025.155363
28. Medlej K, Kazzi AA, El Hajj Chehade A, et al. Complications from Administration of Vasopressors Through Peripheral Venous Catheters: An Observational Study. *J Emerg Med*. Jan 2018;54(1):47–53. doi:10.1016/j.jemermed.2017.09.007
29. Messina A, Milani A, Morenghi E, et al. Norepinephrine Infusion in the Emergency Department in Septic Shock Patients: A Retrospective 2-Years Safety Report and Outcome Analysis. *Int J Environ Res Public Health*. Jan 19 2021;18(2)doi:10.3390/ijerph18020824
30. Munroe ES, Heath ME, Eteer M, et al. Use and Outcomes of Peripheral Vasopressors in Early Sepsis-Induced Hypotension Across Michigan Hospitals: A Retrospective Cohort Study. *Chest*. Apr 2024;165(4):847–857. doi:10.1016/j.chest.2023.10.027
31. Munroe ES, Co IN, Douglas I, et al. Peripheral Vasopressor Use in Early Sepsis-Induced Hypotension. *JAMA Network Open*. 2025;8(8):e2529148–e2529148. doi:10.1001/jamanetworkopen.2025.29148
32. Nguyen TT, Surrey A, Barmaan B, et al. Utilization and extravasation of peripheral norepinephrine in the emergency department. *Am J Emerg Med*. Jan 2021;39:55–59. doi:10.1016/j.ajem.2020.01.014
33. Padmanaban A, Venkataraman R, Rajagopal S, Devaprasad D, Ramakrishnan N. Feasibility and Safety of Peripheral Intravenous Administration of Vasopressor Agents in Resource-limited Settings. *J Crit Care Med (Targu Mures)*. Oct 2020;6(4):210–216. doi:10.2478/jccm-2020-0030
34. Pancaro C, Shah N, Pasma W, et al. Risk of Major Complications After Perioperative Norepinephrine Infusion Through Peripheral Intravenous Lines in a Multicenter Study. *Anesth Analg*. Oct 2020;131(4):1060–1065. doi:10.1213/ane.0000000000004445
35. Petros A, Melkie A, Kotiso KS, et al. Peripheral line for vasopressor administration: Prospective multicenter observational cohort study for survival and safety. *PLoS One*. 2025;20(10):e0333275. doi:10.1371/journal.pone.0333275
36. Powell SM, Faust AC, George S, Townsend R, Eubank D, Kim R. Effect of Peripherally Infused Norepinephrine on Reducing Central Venous Catheter Utilization. *J Infus Nurs*. Jul–Aug 01 2023;46(4):210–216. doi:10.1097/nan.0000000000000508
37. Prasanna N, Yamane D, Haridasa N, Davison D, Sparks A, Hawkins K. Safety and efficacy of vasopressor administration through midline catheters. *J Crit Care*. Feb 2021;61:1–4. doi:10.1016/j.jcrc.2020.09.024
38. Putland M, Kerr D, Kelly AM. Adverse events associated with the use of intravenous epinephrine in emergency department patients presenting with severe asthma. *Ann Emerg Med*. Jun 2006;47(6):559–63. doi:10.1016/j.annemergmed.2006.01.022
39. Ramanan M, Apte Y, Watts S, et al. A randomised, controlled, feasibility trial comparing vasopressors infused via peripheral cannula versus central venous access for critically ill adults: The VIPCA trial. *Crit Care Resusc*. Jun 2025;27(2):100106. doi:10.1016/j.ccrj.2025.100106
40. Ruchti VE, Wibrow BA, Seet J, Jacques A, Jha N, Anstey MH. A prospective comparison of
© 2026 ZhangJian SJ et al. *JAMA Network Open*.

peripheral metaraminol versus dilute noradrenaline in the intensive care unit. *Anaesth Intensive Care*. Mar 2021;49(2):144–146. doi:10.1177/0310057x20984794

41. Sardaneh AA, Penm J, Oliver M, Gattas D, McLachlan AJ, Patanwala AE. Pharmacoepidemiology of metaraminol in critically ill patients with shock in a tertiary care hospital. *Aust Crit Care*. Nov 2021;34(6):573–579. doi:10.1016/j.aucc.2021.01.002

42. Schmucki R, Rüst CA, Filipovic M. Intra-operative norepinephrine via peripheral venous catheter is safe: A short scientific report. *Eur J Anaesthesiol*. Feb 1 2025;42(2):172–173. doi:10.1097/eja.0000000000002080

43. Shyu D, Ingraham NE, Linke CA, et al. Overview of Peripheral Vasopressor Use in an Academic Health System. *Ann Am Thorac Soc*. Aug 2025;22(8):1201–1209. doi:10.1513/AnnalsATS.202411-1135OC

44. Spiegel RJ, Eraso D, Leibner E, Thode H, Morley EJ, Weingart S. The Utility of Midline Intravenous Catheters in Critically Ill Emergency Department Patients. *Ann Emerg Med*. Apr 2020;75(4):538–545. doi:10.1016/j.annemergmed.2019.09.018

45. Stolz A, Efendy R, Apte Y, Craswell A, Lin F, Ramanan M. Safety and efficacy of peripheral versus centrally administered vasopressor infusion: A single-centre retrospective observational study. *Aust Crit Care*. Sep 2022;35(5):506–511. doi:10.1016/j.aucc.2021.08.005

46. Vitharana HS, Amarasena R. Study on Ward-Based Practice of Vasopressor Administration for Patients with Sepsis, in National Hospital of Sri Lanka (NHSL). Article. *Sri Lankan Journal of Anaesthesiology*. 2023;31(2):130–135. doi:10.4038/slja.v31i2.9144

47. Yasuda H, Rickard CM, Marsh N, et al. Risk factors for peripheral intravascular catheter-related phlebitis in critically ill patients: analysis of 3429 catheters from 23 Japanese intensive care units. *Ann Intensive Care*. Apr 8 2022;12(1):33. doi:10.1186/s13613-022-01009-5

48. Yerke JR, Mireles-Cabodevila E, Chen AY, et al. Peripheral Administration of Norepinephrine: A Prospective Observational Study. *Chest*. Feb 2024;165(2):348–355. doi:10.1016/j.chest.2023.08.019

49. Zichichi A, Wallace R, Daniell J, Rouse G, Ahearn P, Ammar M. Safety of Peripherally Infused Sympathomimetic Vasopressors in the Intensive Care Unit and Emergency Department. *Ann Pharmacother*. Oct 16 2024:10600280241284796. doi:10.1177/10600280241284796

© 2026 ZhangJian SJ et al. *JAMA Network Open*.
