# Supplementary material for: Incidence of Adverse Events in Peripheral Intravenous Vasopressor Use: A Systematic Review and Meta-Analysis
Source: JAMA Netw Open. 2026 Mar 16;9(3):e260710. doi: 10.1001/jamanetworkopen.2026.0710 (PMC12993702; doi:10.1001/jamanetworkopen.2026.0710)
Supplement: Supplement 2. — Data Sharing Statement [file jamanetwopen-e260710-s002.pdf]

Data Sharing Statement

ZhangJian. Incidence of Adverse Events in Peripheral Intravenous Vasopressor Use. *JAMA Netw Open*. Published March 16, 2026. doi:10.1001/jamanetworkopen.2026.0710

Data
Data available: No
